# ACP-CapsPred: an explainable computational framework for identification and functional prediction of anticancer peptides based on capsule network

**DOI:** 10.1093/bib/bbae460

**Published:** 2024-09-18

**Authors:** Lantian Yao, Peilin Xie, Jiahui Guan, Chia-Ru Chung, Wenyang Zhang, Junyang Deng, Yixian Huang, Ying-Chih Chiang, Tzong-Yi Lee

**Affiliations:** Kobilka Institute of Innovative Drug Discovery, School of Medicine, The Chinese University of Hong Kong, 2001 Longxiang Road, Shenzhen 518172, China; School of Science and Engineering, The Chinese University of Hong Kong, 2001 Longxiang Road, Shenzhen 518172, China; Kobilka Institute of Innovative Drug Discovery, School of Medicine, The Chinese University of Hong Kong, 2001 Longxiang Road, Shenzhen 518172, China; School of Science and Engineering, The Chinese University of Hong Kong, 2001 Longxiang Road, Shenzhen 518172, China; Kobilka Institute of Innovative Drug Discovery, School of Medicine, The Chinese University of Hong Kong, 2001 Longxiang Road, Shenzhen 518172, China; School of Medicine, The Chinese University of Hong Kong, 2001 Longxiang Road, Shenzhen 518172, China; Department of Computer Science and Information Engineering, National Central University, 300 Zhongda Road, Taoyuan 320317, Taiwan; School of Medicine, The Chinese University of Hong Kong, 2001 Longxiang Road, Shenzhen 518172, China; School of Medicine, The Chinese University of Hong Kong, 2001 Longxiang Road, Shenzhen 518172, China; School of Medicine, The Chinese University of Hong Kong, 2001 Longxiang Road, Shenzhen 518172, China; Kobilka Institute of Innovative Drug Discovery, School of Medicine, The Chinese University of Hong Kong, 2001 Longxiang Road, Shenzhen 518172, China; School of Medicine, The Chinese University of Hong Kong, 2001 Longxiang Road, Shenzhen 518172, China; Institute of Bioinformatics and Systems Biology, National Yang Ming Chiao Tung University, 1001 Daxue Road, Hsinchu 300093, Taiwan; Center for Intelligent Drug Systems and Smart Bio-devices (IDS2B), National Yang Ming Chiao Tung University, 1001 Daxue Road, Hsinchu 300093, Taiwan

**Keywords:** cancers, anticancer peptides, drug discovery, bioinformatics, capsule networks, sequence analysis

## Abstract

Cancer is a severe illness that significantly threatens human life and health. Anticancer peptides (ACPs) represent a promising therapeutic strategy for combating cancer. *In silico* methods enable rapid and accurate identification of ACPs without extensive human and material resources. This study proposes a two-stage computational framework called ACP-CapsPred, which can accurately identify ACPs and characterize their functional activities across different cancer types. ACP-CapsPred integrates a protein language model with evolutionary information and physicochemical properties of peptides, constructing a comprehensive profile of peptides. ACP-CapsPred employs a next-generation neural network, specifically capsule networks, to construct predictive models. Experimental results demonstrate that ACP-CapsPred exhibits satisfactory predictive capabilities in both stages, reaching state-of-the-art performance. In the first stage, ACP-CapsPred achieves accuracies of 80.25% and 95.71%, as well as F1-scores of 79.86% and 95.90%, on benchmark datasets Set 1 and Set 2, respectively. In the second stage, tasked with characterizing the functional activities of ACPs across five selected cancer types, ACP-CapsPred attains an average accuracy of 90.75% and an F1-score of 91.38%. Furthermore, ACP-CapsPred demonstrates excellent interpretability, revealing regions and residues associated with anticancer activity. Consequently, ACP-CapsPred presents a promising solution to expedite the development of ACPs and offers a novel perspective for other biological sequence analyses.

## Introduction

Cancer, a complex and multifaceted group of diseases characterized by uncontrolled cell growth and proliferation, poses a formidable challenge to public health worldwide [1]. As a leading cause of morbidity and mortality, cancer exerts a profound impact on the overall well-being of populations and places a substantial burden on healthcare systems. Current reports for 2023 indicate over 1.9 million new cancer cases and more than 600 000 cancer-related deaths in the United States alone [2].

The development of anticancer drugs has undergone extensive historical research. Conventional therapies, such as chemotherapeutic drugs, exhibit low specificity, inducing severe side effects and toxicities [3]. Notably, the design of chemotherapeutic drugs lacks discrimination, impacting not only cancer cells but also normal cells, exemplified by the unintended targeting of the intestinal epithelium [4]. Due to the limitations of conventional drugs, recent years have witnessed a surge in research focused on alternative cancer therapies, with a particular emphasis on peptide-based therapy [5]. Peptide drugs such as antimicrobial peptides (AMPs), composed of amino acids linked by peptide bonds, possess unique advantages, including high specificity, low toxicity to normal tissues, bloodstream and non-target tissues cleanliness, low immunogenicity, and excellent solubility [6, 7]. Anticancer peptides (ACPs) constitute a subset of AMPs, representing a promising class of bioactive molecules with significant potential in the field of oncology. ACPs, comprising 10–50 amino acids, inhibit tumour cell proliferation, migration, or angiogenesis, with a lower likelihood of inducing drug resistance. The mechanisms through which ACPs function are diverse, encompassing direct interactions with cancer cell membranes, interference with intracellular signaling pathways, induction of apoptosis, and modulation of the tumour microenvironment. Their multifaceted modes of action make them particularly attractive for therapeutic development, as they offer the potential to overcome issues such as drug resistance and off-target effects commonly associated with traditional cancer treatments. The efficiency of peptide-based cancer vaccines in clinical settings attests to the promise of ACPs in cancer treatment [8, 9]. However, traditional wet laboratory-based approaches for identifying ACPs require a substantial investment of human and material resources. Therefore, an increasing amount of research has been directed toward the utilization of computational methods for ACP identification in recent years.

Machine learning methods such as support vector machine (SVM) and random forest (RF) are commonly used for ACP identification. For instance, Tyagi *et al*. introduced AntiCP, a model utilizing SVM with amino acid composition (AAC) and binary profiles (BP) as features to predict and discover novel ACPs, which demonstrates high accuracy, underscoring its effectiveness[10]. Building upon AntiCP, Agrawal *et al.* extended the capabilities with AntiCP 2.0, incorporating additional features such as dipeptide composition (DPC) and terminus composition (TC) for enhanced ACP identification [11]. In a similar vein, Schaduangrat *et al.* proposed ACPred, a classifier for predicting and characterizing the anticancer activities of peptides. This model integrated various features, including AAC, DPC, physicochemical properties (PHYC), pseudo amino acid composition (PAAC), and amphiphilic pseudo amino acid composition (Am-PAAC), thereby improving the predictive performance and augmenting the interpretability of the model [12]. Liang *et al.* developed a classifier named iACP-GE, which utilizes sequence features of peptides. Feature selection is performed through Gradient Boosting Decision Trees (GBDT), and identification of ACP is accomplished using the Extra Tree (ET) classifier [13]. Arif *et al.* introduced StackACPred, a stacking framework for identifying ACPs, which integrates three nominal feature encoding strategies, N-Segmentation position-specific scoring matrix (N-SegPSSM), pseudo (PsePSSM), and extended PAAC, and employs SVM recursive feature elimination and correlation bias reduction (SVM-RFE + CBR) algorithms for feature optimization[14]. In our earlier research, we introduced a classifier known as GRDF [15], which integrates graphical features from peptide sequences. This classifier seamlessly incorporates evolutionary and composition information of peptides. Moreover, it employs a deep forest algorithm for model construction, enabling precise prediction of ACP. Deng *et al.* proposed ACP-MLC, a two-level prediction framework for identifying ACPs and multi-label classification of their functional types, which employs sequence profiles and physicochemical properties of peptides and utilizes RF to construct classifiers [16]. Deep learning methods have gained popularity in recent years, including Recurrent Neural Network (RNN) and Convolutional Neural Network (CNN). Yu *et al.* implemented DeepACP, a deep learning-based model, systematically exploring convolutional, recurrent, and convolutional-recurrent networks for distinguishing ACPs from non-ACPs. Their findings indicated that RNN with bidirectional long short-term memory (Bi-LSTM) cells outperformed the other architectures [17]. Ahmed *et al.* proposed a novel multi-head CNN architecture called ACP-MHCNN for extracting and combining the sequential, physicochemical, and evolutionary features to identify potential ACPs [18]. The summary of studies on ACP identification is presented in Table 1.

**Table 1:** Summary of existing tools for ACP identification

Method	Feature encoding	Algorithm	Year	Reference
AntiCP	AAC, BP	SVM	2013	[10]
iACP-GAEnsC	Am-PAAC, g-Gap dipeptide composition, Reduce amino acid alphabet composition	Genetic algorithm-based ensemble learning	2017	[19]
ACPred	AAC, DPC, PHYC, PAAC, Am-PAAC	SVM, RF	2019	[12]
AntiCP 2.0	AAC, DPC, TC, BP	SVM	2020	[11]
DeepACP	Amino acid embedding	RNN	2020	[17]
cACP	Quasi-sequence order, Conjoint triad feature, Geary autocorrelation descriptor	SVM	2020	[20]
cACP-2LFS	K-space amino acid pair	SVM	2020	[21]
ACP-MHCNN	Sequential features, PHYC, evolutionary information	CNN	2021	[18]
iACP-GE	BC, BLOSUM62, EGAAC, DMACA	GBDT, ET	2022	[13]
StackACPred	N-SegPSSM, PsePSSM, PAAC	SVM-RFE+CBR, LightGMB, stacking-based ensemble learning	2022	[14]
cACP-DeepGram	Word embedding	Deep neural network	2022	[22]
GRDF	Graphical features of peptides, evolutionary information, BP	Deep forest	2023	[15]
ACP-MLC	AAC, BPF, DDE, TPC, AAINEX, C/T/D	RF	2023	[16]
CAPTURE	Correlational information, distributional information, compositional information, transitional information	Adaboost	2024	[23]
ANNprob-ACPs	AAC, Cross-covariance, DPC, PAAC, Quasi-sequence-order, CTDC, CTDT, word2vector, CKSAAGP	Artificial neural network	2024	[24]
ACP-ML	DPC, PAAC, CTDC, CTDT, CS-Pse-PSSM	Voting-based ensemble learning	2024	[25]

Despite the commendable performance achieved by these methods, they still exhibit some limitations, highlighting the imperative for further research in this domain.

(i) Despite the series of successes achieved by CNNs in recent years, they are not devoid of limitations. Specifically, while CNNs excel in capturing the local features of objects, they exhibit a deficiency in grasping the hierarchical structure of objects. Conventional CNNs face challenges in effectively capturing the hierarchical structure and part-whole relationships intrinsic to objects, resulting in limitations and constraints on comprehending the relationship between the overall and local features of objects.
(ii) These methods, particularly those based on deep learning, often lack interpretability, rendering the constructed models as ”black boxes.” This hinders researchers from comprehending the crucial regions and internal features related to ACPs.
(iii) With the exception of ACP-MLC, all methods have been primarily focused on classifying ACP and non-ACP without delving into the exploration of the specific activity of ACP against distinct cancer types.

To address these challenges, we propose a two-stage computational framework in this study, named ACP-CapsPred, designed to identify ACPs and their functional activities against various cancer types. The first stage of ACP-CapsPred is dedicated to discriminating between ACP and non-ACP, while the second stage focuses on predicting the functional activity of ACP against different cancer types. The first-stage model trained on the datasets from prior studies, while the second stage incorporates manually curated datasets comprising ACPs exhibiting anticancer activity against five specific cancer types. We integrate a protein language model for extracting residue embeddings, incorporating evolutionary information and physicochemical properties of peptide sequences to construct comprehensive peptide profiles. Moreover, to overcome the limitations of traditional CNNs and enhance model interpretability, ACP-CapsPred employs a next-generation neural network, the capsule network. In contrast to conventional neural networks, capsule networks introduce the concept of ”capsules,” representing patterns using vectors where the length of the vector denotes the probability of pattern existence. Our past research has demonstrated the effectiveness of capsule networks in predicting the functional activities of peptide sequences [26]. Capsule networks excel at the spatial modeling of objects, effectively capturing hierarchical relationships within objects. Experimental results demonstrate that both stages of ACP-CapsPred yield satisfactory predictive outcomes, achieving state-of-the-art performance. ACP-CapsPred holds significant promise for advancing anticancer drug development and offers novel perspectives for other biological sequence analysis tasks.

## Materials and methods

### Dataset preparation

To make a fair comparison with the previous methods, in the first phase of the task, which is to distinguish between ACP and non-ACP, we selected two benchmark datasets from previous works, Set 1 and Set 2 [15]. Both datasets have positive samples that are experimentally ACPs, but the negative samples are different. The negative samples in Set 1 are antimicrobial peptides that do not possess anticancer activity. The negative samples in Set 2 are randomly selected peptides from Swiss-Prot. These two datasets were subsequently split into train and test sets in an 8:2 ratio. The train set was used to fit the model and tune its hyperparameters through five-fold cross-validation. The test set was used to evaluate the final model. The sizes of these two datasets are shown in Table 2.

**Table 2:** Overview of two datasets in the first stage

Dataset	Train/Test	Positive	Negative	Total
Set 1$^{a}$	Train Set	632	641	1,273
	Test Set	161	158	319
Set 2$^{b}$	Train Set	716	677	1,393
	Test Set	186	170	356

$^{a}$ The positive samples in Set 1 are experimentally validated ACPs. The negative samples in Set 1 are AMPs but do not possess anticancer activity. $^{b}$ The positive samples in Set 2 are experimentally validated ACPs. The negative samples in Set 2 are random peptides, which are assumed to be non-ACPs.

In the second stage of ACP-CapsPred, which involves predicting the functional activities of ACP against various cancers, we selected five categories, namely breast, lung, colon, cervical, and skin, from the CancerPPD database [27], which is a comprehensive repository of experimentally validated ACPs and proteins. Within CancerPPD, ACPs are categorized into 21 classes based on tissue, and we opted for categories with a substantial count of unique peptides exceeding 200 entries to serve as our classification labels. Given that ACPs typically exhibit lengths ranging from 10 to 50 residues, we further excluded entries with lengths less than 10. The size of the datasets in the second stage is outlined in Table S1.

### Overall framework of ACP-CapsPred

**Figure 1:**
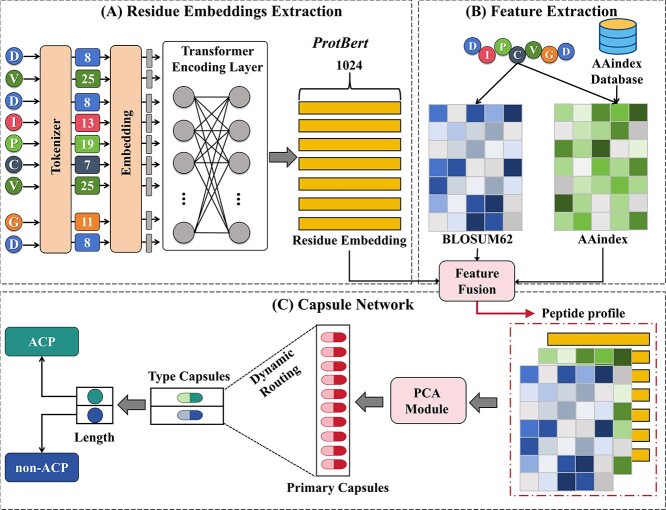
The architecture of ACP-CapsPred; ACP-CapsPred consists of three modules, namely (A) Residue Embedding Module, (B) Feature Extraction Module, and (C) Capsule Network Module; the residue embedding module integrates pretrained protein language models to obtain effective representations of peptide residues, and the feature extraction module combines the BLOSUM62 matrix and AAindex database for peptides to capture evolutionary information and physicochemical properties; residue embedding, BLOSUM62 matrix, and AAindex matrix collectively constitute the peptide profile, and the capsule network module receives the peptide profile, initially undergoes dimensionality reduction using PCA, and subsequently processes and explores the feature matrix for the ultimate classification of ACP and non-ACP.

The workflow of ACP-CapsPred is depicted in Fig. 1, comprising three main modules: residue embedding extraction module, feature extraction module, and capsule network module. Initially, ACP-CapsPred integrates a pretrained protein language model, ProtTrans[28], to extract contextual semantic information, representing each amino acid as a 1024-dimensional embedding. In the feature extraction module, ACP-CapsPred extracts evolutionary information of peptide sequences using the BLOcks SUbstitution Matrix 62 (BLOSUM62). Additionally, ACP-CapsPred incorporates the amino acid index (AAindex) database [29] to capture the physicochemical properties of amino acid sequences. Residue embeddings, BLOSUM62 matrix, and AAindex matrix together constitute comprehensive profiles of peptides. Subsequently, Principal Component Analysis (PCA) [30] is applied to reduce the dimensionality of the extracted feature matrix. The reduced features are then input into the capsule network module for further feature exploration, culminating in the classification of ACP and non-ACP.

### Residue embedding extraction

In recent years, large-scale language models have offered new insights into natural language processing (NLP) techniques. Researchers have proposed numerous protein language models based on amino acid sequences, enabling the extraction of contextual semantic information, thus facilitating the retrieval of discriminative features and enhancing model performance [28, 31, 32].

In NLP tasks, Bidirectional Encoder Representations from Transformers (BERT) architecture [33] is typically employed to extract information from sequences. The transformer architecture relies on self-attention mechanisms [34] to automatically capture relationships between all possible amino acid pairs within a sequence, thereby enhancing representational capacity. In this study, we employed ProtBert [28], a self-supervised pre-trained model based on BERT, to extract contextual semantic information from peptide sequences.

ProtBert is pretrained using the BFD dataset [35], which encompasses 393 billion amino acids from 2.1 billion protein sequences. As illustrated in Fig. 1, each amino acid in every input sequence is initially encoded into numerical tokens by a tokenizer. These tokens are subsequently fed into a transformer architecture composed of 30 hidden layers, with each hidden layer consisting of 16 self-attention heads. Ultimately, each residue is represented as a 1024-dimensional embedding.

### Feature extraction

Prior research has demonstrated that incorporating evolutionary information from proteins enhances the performance of protein sequence analysis tasks, particularly in the context of proteins exhibiting low sequence similarity [36–38]. BLOSUM62 is a fundamental and widely utilized tool in bioinformatics. This substitution matrix plays a pivotal role in quantifying the similarity between amino acid sequences, facilitating the comparison of proteins and the identification of conserved regions across diverse biological entities. The scores within the BLOSUM62 matrix signify the probability of amino acid substitution by another amino. When considering the representation of a peptide (or protein) sequence $S$ with a length of $L$, each constituent residue is denoted by the corresponding row within this matrix, effectively representing a $20$-dimensional vector for each residue. Consequently, sequence $S$ can be succinctly represented as an $L\times 20$-dimensional matrix, as depicted below 


(1) \begin{align*} \mathrm{BLOSUM62} &= \left[\begin{array}{@{}cccc@{}} p_{1,1} & p_{1,2} & \ldots & p_{1,20}\\ p_{2,1} & p_{2,2} & \ldots & p_{2,20}\\ \vdots & \vdots & \ddots & \vdots \\ p_{L,1} & p_{L,2} & \ldots & p_{L,20} \end{array} \right]\end{align*}

where $p_{n,i}$ denotes the likelihood of $n$th amino acids within a peptide being replaced by another amino acid.

In addition to evolutionary information, incorporating the physicochemical properties of amino acids also contributes to protein sequence analysis tasks [15,38,39]. Therefore, in this study, we have integrated the AAindex matrix, which is a fundamental resource in the field of bioinformatics, standing as a comprehensive and systematically curated database of amino acid indices [29]. These indices encapsulate various physicochemical and biochemical properties associated with amino acids. AAIndex is instrumental in bridging the gap between the biological sequences of proteins and their structural and functional characteristics.

Currently, the AAindex database comprises 531 distinct physicochemical properties, which are summarized in the supplementary material. In the context of a peptide sequence $S$, which has a length of $L$, each amino acid is encoded as a $531$-dimensional vector. Within this vector, every numerical value represents a specific physicochemical property. Consequently, the peptide sequence $S$ can be encoded as an $L\times 531$-dimensional matrix, as depicted below. 


(2) \begin{align*} \mathrm{AAindex} &= \left[ \begin{array}{@{}cccc@{}} a_{1,1} & a_{1,2} & \ldots & a_{1,531}\\ a_{2,1} & a_{2,2} & \ldots & a_{2,531}\\ \vdots & \vdots & \ddots & \vdots \\ a_{L,1} & a_{L,2} & \ldots & a_{L,531}, \end{array} \right]\end{align*}

where $a_{n,i}$ refers to the $i$th physicochemical property of the $n$th amino acid.

By incorporating residue embedding, the BLOSUM62, and AAindex matrices, each residue is encoded as a $1575$-dimensional vector. A peptide sequence of length $L$ is then encoded as an $L\times 1575$-dimensional comprehensive numerical matrix. We employed PCA for dimensionality reduction to mitigate overfitting and enhance training efficiency. PCA is a fundamental dimensionality reduction technique extensively employed in various scientific domains, particularly in multivariate data analysis, statistics, and machine learning. It aims to uncover the underlying structure or patterns in high-dimensional data by transforming it into a new coordinate system. PCA allows for efficient data representation with reduced dimensionality while retaining as much relevant information as possible. After dimension reduction, a multi-peptide sequence $S$ with a length of $L$ is ultimately represented as an $L\times 256$-dimensional feature matrix.

### Capsule network

**Figure 2:**
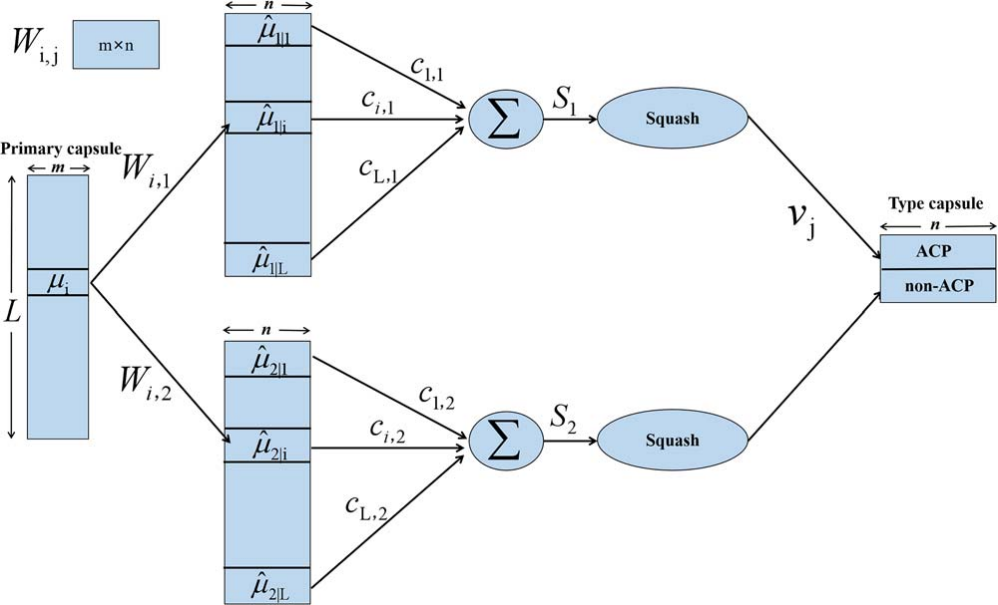
The computational process between primary capsules and type capsules.

Unlike traditional neural networks, capsule networks employ vector-format inputs and outputs [40, 41]. Each capsule represents a distinct pattern, with each dimension of the capsule signifying a unique feature of that pattern, and the length of the capsule indicating the probability of that pattern’s presence. Typically, Capsule networks are composed of primary capsule layers and type capsule layers. The number of type capsules corresponds to the total categories for final classification labels. In the first stage of ACP-CapsPred, the objective is to distinguish between ACP and non-ACP, which is a binary classification task. Hence, the number of type capsules is set to 2. The computational process between the primary capsule layer and the type capsule layer is illustrated in Fig. 2.

The primary capsule layer $u_{i}$ initially undergoes multiplication with weight matrices $W_{1}$ and $W_{2}$, yielding intermediate vectors $\hat{u}_{1 \mid i}$ and $\hat{u}_{2 \mid i}$ corresponding to the two distinct classification categories. The predictive vectors $S_{j}$, which represent ACP and non-ACP, are obtained through a weighted sum over all $\hat{u}_{j \mid i}$ and coupling coefficients $c_{i, j}$, as expressed in the following formula: 


(3) \begin{align*}& S_{j}=\sum_{i=1}^{L} c_{i, j} \hat{u}_{j \mid i},\end{align*}

where $L$ denotes the number of primary capsules.

The coupling coefficients ($c_{i, j}$) delineate the likelihood of establishing connections between the $i$th primary capsule and the $j$th type capsule. The cumulative value of $c_{i, j}$ across two type capsules is equal to 1, i.e. $\sum _{i=1}^{L} \sum _{j=1}^{2} c_{i,j} = 1$. Coupling coefficients are determined by the dynamic routing algorithm; for details on the dynamic routing algorithm refer to the **Supplementary material**.

In accordance with the concept of capsule networks, the length of a capsule signifies the likelihood of a specific category’s occurrence. A non-linear function known as Squash operates on the prediction vector $S$ to obtain the type capsules ($V_{j}$), with the Squash function defined as follows: 


(4) \begin{align*}& \textrm{ Squash}\ (\mathrm{S})=\frac{\|\mathrm{S}\|^{2}}{1+\|\mathrm{S}\|^{2}} \frac{\mathrm{S}}{\|\mathrm{S}\|}.\end{align*}

Utilizing the Squash function, the length of vector $S$ is compressed within the range of 0 to 1 while preserving its direction. The length of the type capsule indicates the probability associated with the respective category. Consequently, the $L2$ norms of the type capsules are computed individually to derive the final prediction probabilities, which are shown below. 


(5) \begin{align*}& p_{j}=\left\|v_{j}\right\|_{2}.\end{align*}

Following the work of Sabour *et al.*[40], we have employed margin loss as the model’s loss function, as illustrated below 


(6) \begin{align*}& L_{c}=I_{c} \max \left(0, m^{+}-\left\|v_{c}\right\|_{2}\right)^{2}+\lambda\left(1-I_{c}\right) \max \left(0,\left\|v_{c}\right\|_{2}-m^{-}\right)^{2},\end{align*}

where $c$ and $I$ denote the classification category and the binary indicator function, respectively. When a sample belongs to category c, $I_{c}$ equals 1; otherwise, $I_{c}$ equals 0. For the hyperparameters $\mathrm{m}^{+}, \mathrm{m}^{-}$, and $\lambda $, we have adopted the recommended values, which are 0.9, 0.1, and 0.5, respectively.

### Functional prediction of ACP against various cancers

**Figure 3:**
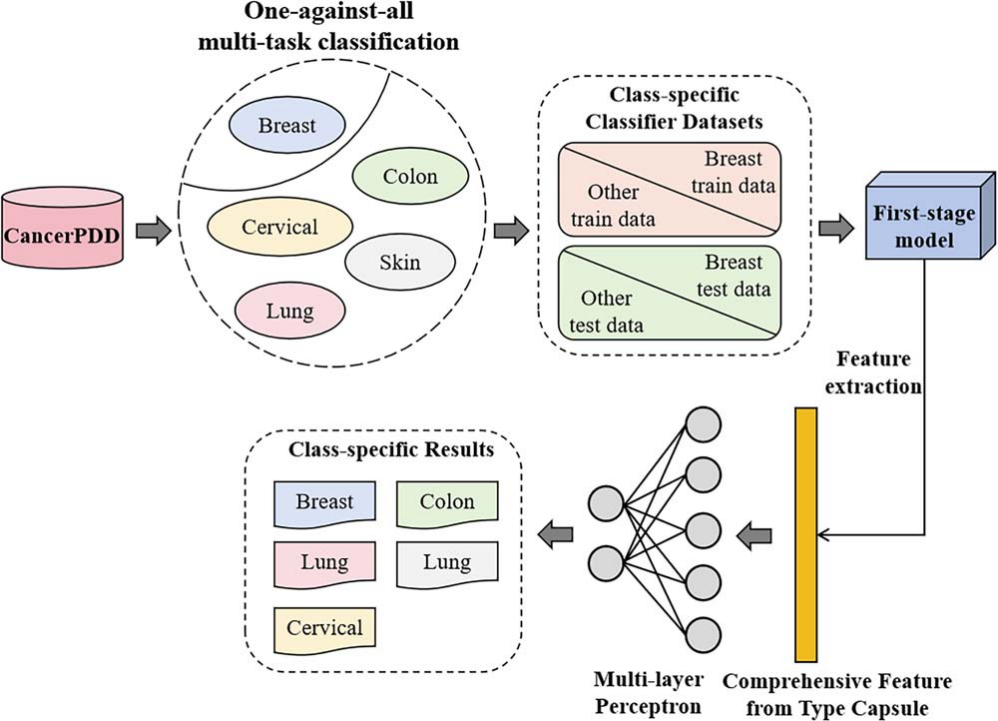
The functional prediction workflow for ACP against various cancers in the second stage; due to the potential activity of certain peptides against one or multiple types of cancers, we have established a one-against-all multi-task classification pipeline, and in this stage, ACP-CapsPred utilizes the model from the first stage to extract features and subsequently employs a multi-layer perceptron for classification.

Capsule networks demonstrate notable advantages in feature processing and exploration, particularly excelling in capturing intricate hierarchical and spatial relationships within data. In the second stage of ACP-CapsPred, the objective is to predict the functional activities of ACP against distinct cancer types. Given the limited number of samples for different labels, we opted not to retrain the model. Instead, we fully harnessed the capsule network’s feature processing capabilities. We leveraged the well-trained model from the first stage to extract discriminative latent features from type capsules for second-stage prediction. Figure 3 illustrates the workflow for this stage.

As mentioned in Section 8, we gathered the five most abundant ACPs with documented anticancer activities targeting breast, colon, cervical, lung, and skin cancers from CancerPPD. Due to the potential activity of certain sequences against multiple cancers, we employed a one-against-all multi-task learning strategy in the second stage. Subsequently, we established five class-specific classifiers. Sequences exhibiting a specific anticancer activity type were designated as positive instances within the dataset pertaining to the corresponding ACP class classifier. Conversely, sequences not exhibiting the specified activity were categorized as negative instances. For instance, a sequence demonstrating anti-breast activity was considered a positive sample during the construction of the anti-breast classifier but was regarded as a negative sample for the construction of the other classifiers. When constructing class-specific classifiers, ACPs with varying functional activities were input into the model previously trained in the first stage. Subsequently, discriminative features were extracted from the type capsules. Utilizing these features, a multi-layer perceptron was constructed to predict ACP functional activity against specific cancer types.

### Performance assessment

To assess the performance of our proposed approach, we employed several machine learning evaluation metrics, including Accuracy, Precision, Recall, True Positive Rate (TPR), False Positive Rate (FPR), and F1-score. These metrics are defined as follows: 


(7) \begin{align*} Accuracy & = \frac{TP + TN}{TP + TN + FP + FN} \end{align*}

(8) \begin{align*} Precision & = \frac{TP}{TP+FP} \end{align*}

(9) \begin{align*} Recall & = \frac{TP}{TP+FN} = TPR \end{align*}

(10) \begin{align*} FPR & = \frac{FP}{FP+TN} \end{align*}

(11) \begin{align*} F1-score & = \frac{2 \times Precision \times Recall} {Precision + Recall}, \end{align*}

where TP, TN, FP, and FN represent the number of true positives, true negatives, false positives, and false negatives, respectively. The Area Under the Receiver Operating Characteristic Curve (AUROC) and the Area Under the Precision-Recall Curve (AUPRC) are also used to assess the performance in this study.

The models experienced training for 100 epochs to ensure adequate fitting, utilizing the Adam optimizer [42] with an initial learning rate of 0.1. In this study, there are three key hyperparameters: the dimension of primary capsules, the number of routing iterations, and the dimension of type capsules. We employed grid search and cross-validation techniques to explore these hyperparameters, and the specific search space is detailed in Table S2. We systematically evaluated all possible combinations using five-fold cross-validation to determine the optimal hyperparameters that maximize performance. Accuracy served as our criteria. The training curves of ACP-CapsPred on two datasets are shown in Fig. S1. The pipeline of ACP-CapsPred was implemented using PyTorch [43], and the training procedure was executed across 4 $\times $ Nvidia 2080 Ti GPUs.

## Results and discussion

### Performance comparison of ACP-CapsPred with existing methods

We compared ACP-CapsPred with existing ACP prediction methods. In the first stage, which involves distinguishing ACP from non-ACP sequences, ACP-CapsPred achieved state-of-the-art performance, demonstrating precise predictions for ACP, as depicted in Table 3. Their confusion matrices were summarized as shown in Table S3. Specifically, on Set 1, ACP-CapsPred attained the highest Accuracy of 80.25%, surpassing the second-ranked method by 3.1%. Additionally, ACP-CapsPred achieved the highest Precision on Set 1, at 81.70%. Similarly, on Set 2, ACP-CapsPred exhibited outstanding performance with the highest Accuracy and Recall, reaching 95.71% and 95.43%, respectively. Moreover, while ensuring precise predictions for ACP, ACP-CapsPred also achieved more balanced predictions. On both Set 1 and Set 2, ACP-CapsPred achieved the highest F1-score, at 79.86% and 95.90%, respectively. The effectiveness of our proposed approach in precise and balanced ACP prediction can be attributed to several crucial factors.

**Table 3:** Summary of performance comparison with other existing methods in the first stage

Dataset	Method	Accuracy	Precision	Recall (=TPR)	F1-score	FPR
Set 1 ACP versus non-ACP (AMP)	AntiCP	53.29%	52.03%	**95.65%**	67.40%	89.87%
	AntiCP2	70.22%	75.78%	62.25%	67.13%	20.25%
	AMPFun	68.65%	67.84%	72.05%	69.88%	34.81%
	dbAMP	67.40%	63.77%	81.99%	71.74%	47.47%
	ACPred	54.86%	53.23%	86.96%	66.04%	77.85%
	iACP-GE	75.86%	75.30%	77.63%	76.45%	25.95%
	StackACPred	73.04%	75.51%	68.94%	72.07%	22.78%
	DeepACP	57.99%	55.05%	91.30%	68.69%	75.95%
	ACP-MHCNN	57.05%	54.28%	94.40%	68.93%	81.01%
	GRDF	77.12%	76.83%	78.26%	77.54%	24.05%
	ACP-MLC	71.16%	70.32%	83.30%	76.27%	36.08%
	cACP	64.57%	72.50%	62.70%	67.24%	24.05%
	cACP-2LFS	66.77%	68.75%	66.26%	67.48%	31.01%
	cACP-DeepGram	65.20%	58.75%	67.62%	62.87%	48.73%
	iACP-GAEnsC	67.39%	70.00%	66.66%	68.28%	29.11%
	ANNprob-ACPs	73.35%	73.75%	73.35%	73.55%	26.58%
	ACP-ML	75.55%	75.78%	75.78%	75.78%	24.68%
	CAPTURE	75.55%	75.78%	75.78%	75.78%	24.68%
	**This study**	**80.25%**	**81.70%**	78.12%	**79.86%**	**17.61%**
Set 2 ACP versus non-ACP (Random Peptide)	AntiCP	87.92%	**100.00%**	76.88%	86.93%	**0.00%**
	AntiCP2	91.57%	92.39%	91.40%	91.89%	8.24%
	AMPFun	77.25%	89.47%	63.89%	74.61%	8.24%
	dbAMP	49.72%	51.71%	56.99%	54.22%	58.24%
	ACPred	88.48%	89.62%	88.17%	88.89%	11.18%
	iACP-GE	89.60%	92.57%	87.09%	89.75%	7.65%
	StackACPred	92.97%	96.00%	90.32%	93.07%	4.12%
	DeepACP	90.73%	93.60%	87.97%	90.70%	6.47%
	ACP-MHCNN	91.57%	95.34%	88.17%	91.62%	4.71%
	GRDF	94.10%	97.69%	90.86%	94.15%	2.35%
	ACP-MLC	84.83%	89.28%	80.64%	84.74%	10.59%
	cACP	88.28%	86.93%	89.47%	88.18%	14.71%
	cACP-2LFS	85.71%	80.70%	89.03%	84.66%	23.53%
	cACP-DeepGram	87.14%	89.47%	85.00%	87.17%	11.18%
	iACP-GAEnsC	77.14%	90.64%	70.77%	79.48%	8.24%
	ANNprob-ACPs	91.57%	91.94%	91.94%	91.94%	8.82%
	ACP-ML	91.57%	91.94%	91.94%	91.94%	8.82%
	CAPTURE	92.29%	84.57%	92.29%	88.26%	18.24%
	**This study**	**95.71%**	95.98%	**95.43%**	**95.90%**	4.12%

Firstly, ACP-CapsPred integrates residue embedding from the protein language model, which was pretrained on extensive protein datasets. The extracted residue embeddings can learn rich sequence and contextual semantic information from large-scale protein sequence data, helping the model understand relationships between residues, complex interactions, and functional implications. Secondly, ACP-CapsPred incorporates the physicochemical properties of amino acids. By utilizing the AAindex database, we integrate 531 physicochemical properties for each amino acid. Previous research has highlighted the significant utility of physicochemical property information for sequence analysis and multi-omics tasks [39]. Thirdly, this study integrates the evolutionary information of peptide sequences. The BLOSUM62 matrix provides insights into the probability of amino acid substitutions, describing the similarity and dissimilarity of protein sequences. By using the BLOSUM62 matrix, we can more effectively identify conserved regions of protein sequences, thus providing important clues about protein functions. Previous studies have demonstrated the value of integrating evolutionary information for protein function prediction [36, 44]. Compared with our previous research based on capsule networks [26], ACP-CapsPred integrates residue embeddings extracted from protein language models, the physicochemical properties of residues, and evolutionary information to construct a comprehensive profile of peptides, exemplifying its advancement. Lastly, we employ a next-generation neural network, the capsule network, which conducts hierarchical feature learning and models relationships between features, thus providing a more accurate representation of protein sequences [26, 45, 46]. Therefore, the achievement of state-of-the-art performance by ACP-CapsPred is not surprising.

### Performance of functional activity prediction of ACP against various cancers

In the second stage, ACP-CapsPred is employed to predict the functional activity of ACP against various types of cancers. As depicted in Fig. S2, certain ACP sequences exhibit anticancer activity against one or multiple specific cancer types. Consequently, ACP-CapsPred adopts a one-against-all multi-task learning strategy to characterize the functional activity of ACP against different cancer types.

In prior research, ACP-MLC [16] conducted ACP prediction for different types of cancer. Therefore, we considered ACP-MLC as the baseline model, and the comparison results and confusion matrices are shown in Tables 4 and S4, respectively. The results indicate that ACP-CapsPred performs slightly worse than ACP-MLC in predicting ACPs against colon cancer, outperforming ACP-MLC in other cancer types. Across the five cancer types, ACP-CapsPred achieves an average accuracy of 90.75% and an average F1-score of 91.38%. Particularly noteworthy is ACP-CapsPred’s highly accurate prediction of ACPs against breast cancer, reaching an accuracy of 94.59%.

**Table 4:** Summary of performance comparison with other existing methods in the second stage

Cancer type	Method	Accuracy	Precision	Recall (=TPR)	F1-score	FPR
Breast	ACP-MLC	93.33%	92.12%	92.72%	92.42%	13.04%
	**ACP-CapsPred**	**94.59%**	**96.08%**	**96.08%**	**96.08%**	**3.92%**
Lung	ACP-MLC	55.55%	55.55%	**100.00%**	71.42%	85.47%
	**ACP-CapsPred**	**82.43%**	**85.14%**	82.05%	**83.57%**	**11.42%**
Colon	**ACP-MLC**	**91.89%**	**96.07%**	**92.45%**	**94.23%**	10.71%
	ACP-CapsPred	91.60%	90.83%	92.16%	91.49%	**8.92%**
Cervical	ACP-MLC	49.31%	51.35%	50.00%	50.66%	8.33%
	**ACP-CapsPred**	**94.59%**	**92.11%**	**97.22%**	**94.60%**	**2.77%**
Skin	ACP-MLC	71.16%	67.00%	84.47%	74.73 %	41.18%
	**ACP-CapsPred**	**90.54%**	**90.00%**	**92.31%**	**91.14%**	**11.42%**
Average	ACP-MLC	72.25%	72.42%	83.93%	76.69%	31.74%
	**ACP-CapsPred**	**90.75%**	**90.83%**	**91.96%**	**91.38%**	**7.69%**

To further investigate the predictive capability of ACP-CapsPred for different ACP functional activities, we compared it with three common machine learning methods, including RF, SVM, and K-Nearest Neighbors as baseline models. The performance comparison and details of the baseline models are shown in Table S5. ACP-CapsPred outperformed these baseline models, achieving the highest F1-score across the five types of ACP labels. Moreover, ACP-CapsPred demonstrated superior average Accuracy, average Precision, average F1-score, average FPR, average AUROC, and average AUPRC, further confirming its effectiveness. The success of ACP-CapsPred in ACP functional activity prediction can be primarily attributed to the discriminative features extracted by the capsule network, benefiting from its hierarchical feature learning capabilities [47].

### The effectiveness of capsule network architecture

**Figure 4:**
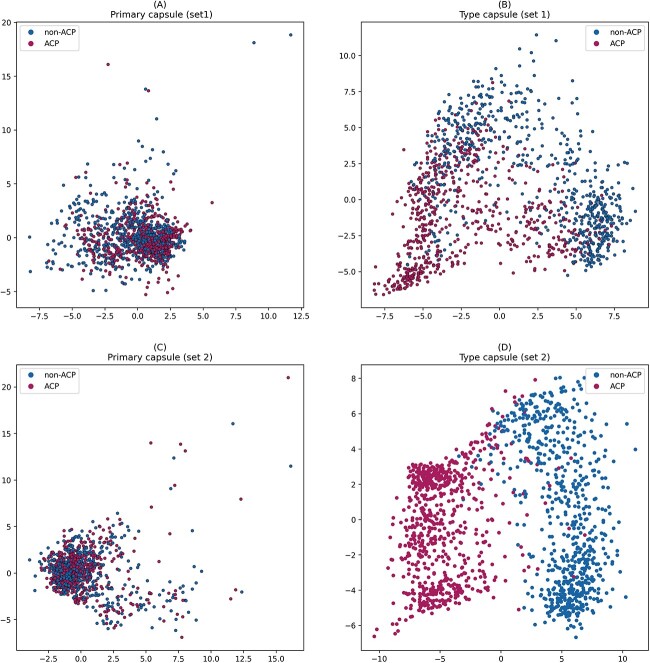
PCA visualization of the feature space; (A) visualization of the primary capsule layer of Set 1; (B) visualization of the type capsule layer of Set 1; (C) visualization of the primary capsule layer of Set 2; (D) visualization of the type capsule layer of Set 2, and for each sample, the capsules from the primary capsule layer and the type capsule layer are concatenated separately and then subjected to PCA for dimensionality reduction to visualize each sample in a 2D space; ACPs and non-ACPs are shown in red color and blue color, respectively.

To validate the effectiveness of the capsule network architecture, we visualized the training samples at the primary capsule layer and the type capsule layer. Specifically, for each sample, we concatenated all capsules from the primary capsule layer and the type capsule layer separately to obtain the latent features for each sample in both layers. Then, the PCA technique was employed for dimensionality reduction, converting the high-dimensional features into two dimensions, as presented in Fig. 4. Figure 4A and B represents scatterplots of samples from Set 1 in the primary capsule layer and the type capsule layer, while Fig. 4C and D represents scatterplots of samples from Set 2 in the primary capsule layer and the type capsule layer. The figures show that ACPs represented by red dots and non-ACPs represented by blue dots cluster together in the primary capsule layer and do not exhibit any distinct distribution. However, ACPs and non-ACPs are separable at the type capsule layer. This is attributed to the capsule network’s ability to perform knowledge processing and hierarchical modeling in the primary capsule layer, ultimately extracting discriminative features [48].

In order to investigate the impact of the capsule network architecture on model performance further, we replaced the capsule network architecture with a multi-layer perceptron. We trained the newly configured model on Sets 1 and 2 and compared its performance with ACP-CapsPred, as summarized in Table 5. The table shows that models without the capsule network architecture exhibit a significant drop in performance compared with ACP-CapsPred. Accuracy decreased by 4.70% and 3.14% on Set 1 and Set 2, respectively. Precision, Recall, and F1-score also showed significant decreases.

**Table 5:** Summary of performance comparison with the model without capsule networks

Dataset	Model	Accuracy	Precision	Recall (=TPR)	F1-score	FPR	AUROC	AUPRC
Set 1	Without Capsule network	75.55%	75.95%	75.50%	75.72%	23.75%	0.863	0.832
	**ACP-CapsPred**	**80.25%**	**81.70%**	**78.12%**	**79.86%**	**17.61%**	**0.942**	**0.947**
Set 2	Without Capsule network	92.57%	94.05%	90.80%	93.30%	5.85%	0.962	0.972
	**ACP-CapsPred**	**95.71%**	**95.98%**	**95.43%**	**95.90%**	**4.12%**	**0.986**	**0.989**

**Figure 5:**
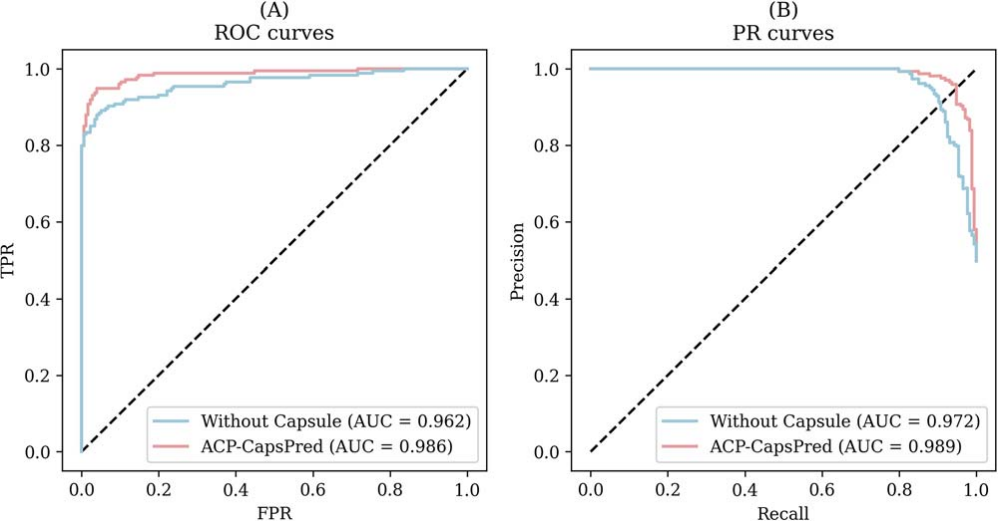
ROC and PR curves for ACP-CapsPred and the model without a capsule network on Set 2.

For a more intuitive performance comparison, we plotted the two models’ ROC and PR curves. The ROC and PR curves for Set 1 and Set 2 are presented in Figs S3 and 5, respectively. Figure 5 shows that the ROC curve representing ACP-CapsPred is positioned in the upper left corner, achieving a larger AUROC (0.986) than the model without the capsule network. Similarly, the PR curve representing ACP-CapsPred is in the upper right corner, achieving a larger AUPRC (0.989) than the model without the capsule network. The comparison with the model without the capsule network further demonstrates the capsule network’s capability to significantly enhance ACP recognition, which can be attributed to several factors. Firstly, compared with traditional deep neural networks, capsule networks utilize vectors to represent inputs and outputs instead of simple scalars. This allows capsule networks to represent entities more richly, thereby enhancing feature representation capabilities and enabling them to excel in understanding complex data structures. Secondly, the dynamic routing mechanism plays a crucial role, enabling different capsules to communicate, negotiate, and reach a consensus. This mechanism helps model the hierarchical relationships between different features, thereby improving the model’s generalization ability and robustness. Given these advantages, capsule networks are beneficial for modeling the hierarchical relationships of different residues in sequences for ACP activity prediction tasks. Therefore, it is understandable that ACP-CapsPred achieves satisfactory performance in this study.

### Comparing the effectiveness of PCA and other dimensionality reduction methods

To comprehensively consider the profile of peptide sequences, ACP-CapsPred integrates residue embeddings, the BLOSUM62 matrix, and the AAindex matrix. However, the large dimension of the feature matrix might make the model difficult to train [49]. Therefore, we incorporated PCA for dimensionality reduction to eliminate redundant information and noise from the feature matrix, further extracting the most representative features. This process reduces the number of features, accelerates the training process, and decreases storage space and computational costs.

We compared PCA with several other commonly used dimensionality reduction techniques, including Factor Analysis, Independent Component Analysis, and UMAP, to validate the effectiveness of PCA. The performance comparison is shown in Table S6, which indicates that models using PCA for dimensionality reduction achieved the best performance, with accuracy improvements of 5% and 2.6% over other methods on the two datasets, respectively. Furthermore, the F1-scores with PCA were 7.2% and 2.8% higher than other dimensionality reduction methods on the two datasets. ACP-CapsPred and the models trained with other dimensionality reduction methods share the same architecture. However, ACP-CapsPred significantly outperforms the others, indicating that the PCA dimensionality reduction strategy used by ACP-CapsPred can extract more representative features, thereby enhancing the model’s predictive performance.

### Ablation experiment

In order to explore the influence of different feature encodings on prediction results, we conducted the following three ablation experiments:

(i) Without BLOSUM62: The feature matrix excludes the BLOSUM62 matrix for amino acid sequences and is composed of the AAindex matrix and embeddings from a pre-trained model.
(ii) Without AAindex: The feature matrix excludes the AAindex matrix for amino acid sequences and is composed of the BLOSUM62 matrix and embeddings from a pre-trained model.
(iii) Without pre-trained model: The feature matrix excludes embeddings from a pretrained model and is composed of the BLOSUM62 matrix and AAindex matrix.

The results of the ablation experiments are presented in Table 6. The predictive model performance is most significantly compromised when the pretrained model is omitted. Particularly, on Set 1, the model without the pretrained model exhibited an accuracy of only 65.52%, representing a decrease of $\sim $14.73% compared with ACP-CapsPred. In contrast, the models without BLOSUM62 and AAindex only exhibited decreases of 5.01% and 2.82%, respectively. Similarly, on Set 2, the model without the pretrained model displayed a decrease in accuracy of 2.74% compared with ACP-CapsPred, while the models without BLOSUM62 and AAindex only exhibited decreases of 1.42% and 2.01%, respectively.

**Table 6:** Summary of performance in ablation experiments

Dataset	Model	Accuracy	Precision	Recall (=TPR)	F1-score	FPR	AUROC	AUPRC
Set 1	Without BLOSUM62	75.24%	74.55%	76.88%	74.89%	26.25%	0.884	0.890
	Without AAindex	77.43%	76.83%	78.75%	77.13%	23.75%	0.909	0.917
	Without the pre-trained model	65.52%	63.74%	**85.16%**	72.91%	48.13%	0.847	0.844
	**ACP-CapsPred**	**80.25%**	**81.70%**	78.12%	**79.86%**	**17.61%**	**0.942**	**0.947**
Set 2	Without BLOSUM62	94.29%	94.25%	94.25%	94.25%	5.85%	0.980	0.983
	Without AAindex	93.70%	92.70%	94.83%	93.75%	7.60%	0.984	0.987
	Without the pre-trained model	92.97%	**96.00%**	90.32%	93.07%	4.19%	0.848	0.864
	**ACP-CapsPred**	**95.71%**	95.98%	**95.43%**	**95.90%**	**4.12%**	**0.986**	**0.989**

**Figure 6:**
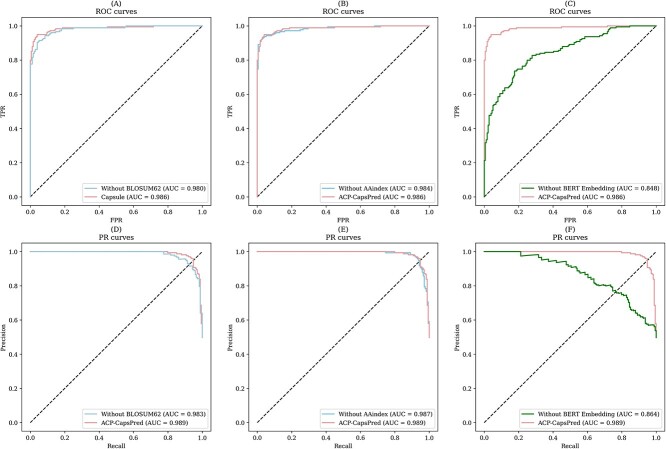
ROC and PR curves of the ablation experiments on Set 2.

To present the results of the ablation experiments more clearly and intuitively, we plotted ROC and PR curves for the three ablation experiments on two datasets, Set 1 and Set 2, as shown in Figs S4 and 6, respectively. Figure 6A–C represents the ROC curves corresponding to the models without BLOSUM62, without AAindex, and without the pretrained model, respectively. It can be observed that the areas enclosed by the ROC curves for the three ablation experiments are reduced compared with ACP-CapsPred. Notably, the ROC curves corresponding to models without BLOSUM62 and without AAindex exhibit a relatively high degree of overlap with the ROC curve corresponding to ACP-CapsPred, and their respective AUROC values are close. This suggests that the impact of BLOSUM62 and AAindex on the model’s predictive performance is relatively small. In contrast, as evident from Fig. 6C, when the model excludes embeddings from the pretrained model, its overall ROC curve shifts lower and to the right, achieving only an AUROC of 0.848, representing a decrease of 0.138 compared with ACP-CapsPred. Figure 6D–F corresponds to the PR curves for the models without BLOSUM62, without AAindex, and without the pretrained model, respectively. Similarly, when the model omits embeddings from the pretrained model, its PR curve shifts lower and to the left, achieving only an AUPRC of 0.864, representing a decrease of 0.125 compared with ACP-CapsPred.

These findings suggest that the embeddings of protein sequences from pretrained protein language models substantially impact model prediction performance. This underscores the notion that semantic features rich in contextual information exert the most significant contribution compared with physicochemical properties and evolutionary information in the context of ACP prediction tasks. Additionally, we compared our encoding approach with the commonly used one-hot encoding strategy, as shown in Table S7. Compared with ACP-CapsPred, the model using one-hot encoding showed 7.5% and 6% lower accuracy on the two datasets, respectively. This demonstrates that the feature encoding method employed by ACP-CapsPred can incorporate peptide information more comprehensively than one-hot encoding.

### Feature analysis

**Figure 7:**
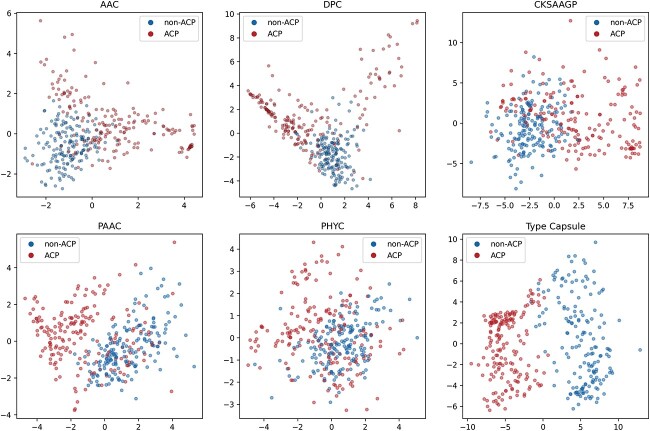
Visualization of positive and negative samples with distinct features is conducted on Set 2; the latent features derived from type capsule of ACP-CapsPred are compared with several widely used hand-craft peptide descriptors, namely AAC, DPC, PAAC, CKSAAGP, and PHYC; the reduction of high-dimensional features to two dimensions is achieved through PCA, and ACPs are represented by red dots, while non-ACPs are represented by blue dots.

The superiority of capsule networks lies in their ability to capture spatial hierarchical relationships among features and more effectively explore features through a dynamic routing mechanism. To visually demonstrate the feature exploration capabilities of capsule networks, we compared features extracted by capsule networks with hand-crafted peptide descriptors, as shown in Fig. 7 for Set 2, respectively. We selected five common hand-crafted peptide descriptors, namely AAC, DPC, CKSAAGP, PAAC, and PHYC, and denoted the features extracted by capsule networks as ”Type Capsule.”

Figure 7 shows that, compared with the five hand-crafted peptide descriptors, the features extracted by capsule networks can categorize ACP and non-ACP into two distinct clusters, allowing for better differentiation. This underscores the capacity of capsule networks to process and explore features at a deeper level, which is critical for the precise prediction of ACP.

### Interpretability of ACP-CapsPred

**Figure 8:**
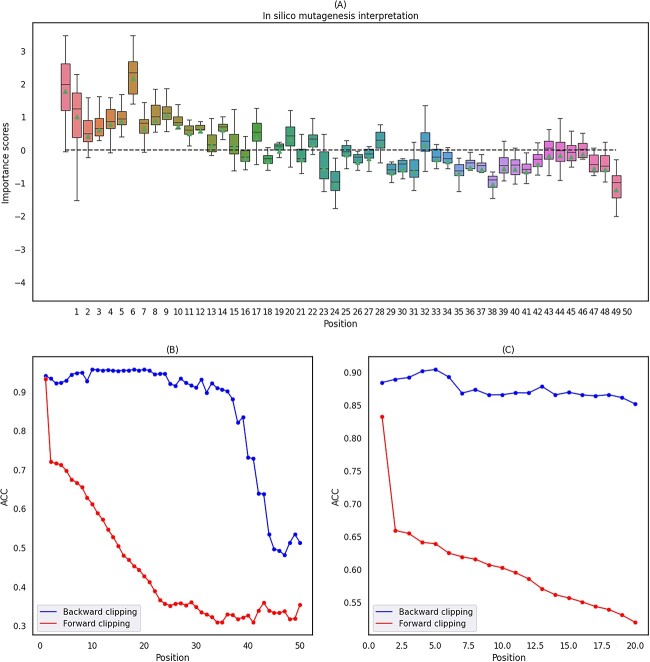
The interpretable analysis on region importance with ACP-CapsPred;(A) the amino acid position importance plot obtained from ISM experiments based on a 50 length ACP; (B) the result of the split experiment based on a 50 length ACP; (C) the result of the split experiment based on all ACP sequences longer than 30 amino, and the forward clipping involves cutting the sequence at the head region while preserving the tail region; conversely, the backward clipping involves cutting the sequence at the tail region while preserving the head region.

In order to investigate the impact of amino acid type and sequence region on the anticancer activity of peptides, we employed an *in silico* mutagenesis (ISM) approach [50] on all sequences in the dataset. Each reference amino acid of the peptide was mutated to 19 other alternative amino acids to evaluate the predicted score changes. The ISM results for an ACP of 50 amino acids in length are depicted in Fig. 8A, indicating that the front portion of the amino acids, particularly the first ten amino acids, significantly influences the prediction scores.

To validate this observation, we conducted clipping experiments on this sequence, and the results are shown in Fig. 8B. We clipped from both the front and rear ends of the sequence and then fed the clipped sequences into the model to assess whether the anticancer activity could be retained. Figure 8B illustrates that a substantial drop in accuracy occurs when the sequence is clipped from the front, especially when the first amino acid is clipped, resulting in about 20% accuracy drop. Conversely, when the amino acids are clipped from the rear, the accuracy remains consistently above 90% as long as fewer than 30 amino acids are removed. This further underscores the significant impact of the front portion of the amino acid sequence on anticancer activity, particularly the first amino acid.

Next, we repeated the aforementioned experiments for all ACP sequences longer than 30 amino acids, sequentially clipping amino acids from both the front and rear. Figure 8C shows the average accuracy obtained from these experiments. Clipping amino acids from the front has a more pronounced impact on their accuracy, especially when the first amino acid is clipped, resulting in an average accuracy decrease of about 20%. Clipping amino acids from the rear has a lesser impact on accuracy.

To further demonstrate the above conclusion, we conducted additional explainable analysis using SHAP values, and the results are shown in Fig. S5. We first extracted feature vectors corresponding to each residue in ACP or non-ACP, followed by summing all the values within the feature vector to obtain the normalized feature for each residue in the sequence, which is illustrated in Fig. S5A and C, where the $x$-axis represents residue positions and the $y$-axis represents different sequences. In this analysis, we randomly selected 50 ACPs and 50 non-ACPs sequences from the test set. Figure S5B and D displays the SHAP values for different residue positions in these ACP and non-ACP sequences, respectively, indicating the impact of residues at different positions on the model output. Specifically, Fig. S5B shows the impact of different residue positions on the model output for the 50 randomly selected ACP sequences, where redder colors indicate a greater contribution to correct ACP predictions. It can be easily observed that residues at the initial positions have a more significant impact, as indicated by the redder colors, which is consistent with Fig. 8. Similarly, Fig. S5D shows the impact of different residue positions on the model output for the 50 randomly selected non-ACP sequences, where bluer colors indicate a greater contribution to correct non-ACP predictions. It is also evident that residues at the initial positions have a more significant impact, as indicated by the bluer colors.

The unique architecture of capsule networks can assist in unraveling the black box of deep learning. To further validate the phenomenon above, we conducted interpretability analysis using capsule networks and generated a heatmap of the importance of amino acid types and positions, as shown in Fig. S6, where darker colors indicate higher importance. Due to variations in the distribution of different sequences, we categorized all sequences into eight groups based on their lengths.

It is readily observable that, within each group, amino acids at the first position exhibit a more significant number of dark cells, indicating that the first position’s amino acid exerts the most significant influence on the anticancer activity of peptides, consistent with the previous conclusion [51]. For example, in sequences of lengths 11 to 15, 16 to 20, and 21 to 25, the amino acid Phenylalanine (F) with hydrophobic properties has the most significant impact at the first position, suggesting a potential link between the anticancer activity of shorter sequences and hydrophobic interactions. In sequences of lengths 26 to 30 and 31 to 35, the amino acid Glycine (G) with polar properties exerts the most significant influence at the first position, implying a strong association between the anticancer activity of longer sequences and electrostatic interactions [51, 52].

**Figure 9:**
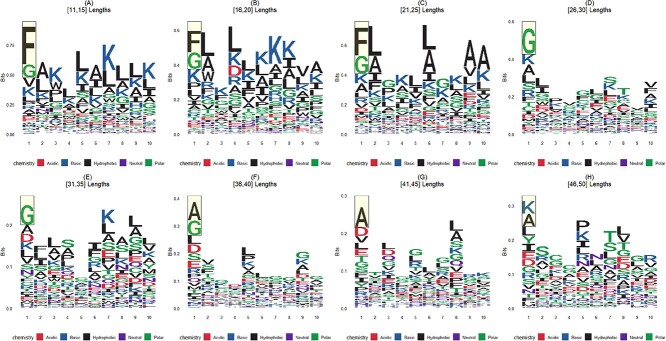
Visualization of frequencies of occurrence regarding first 10 amino acids within ACPs.

Subsequently, we visualized the frequency of occurrence of the first ten amino acids in ACPs within each group and created sequence logos, as shown in Fig. 9. It is evident that in sequences of lengths 11 to 15, 16 to 20, and 21 to 25, amino acids Phenylalanine (F) with hydrophobic properties and Glycine (G) with polar properties have the highest occurrence rates at the first position [53]. In sequences of lengths 26 to 30 and 31 to 35, amino acid G with polar properties has the highest occurrence rate at the first position [54]. In sequences of lengths 36 to 40 and 41 to 45, the amino acid Alanine (A) with hydrophobic properties has the highest occurrence rate [27]. This further underscores that the mechanism of action for ACPs is associated with hydrophobic and electrostatic interactions, consistent with the earlier findings and prior literature[55, 56]. Furthermore, it is observed that for short peptides, hydrophobic interactions may play a dominant role in anticancer activity [57].

## Conclusion

In this study, we proposed a two-stage computational framework, ACP-CapsPred, for predicting ACPs and their targets across various cancers. The first stage of ACP-CapsPred is dedicated to identifying ACPs, while the second stage focuses on predicting the functional activity of ACPs against different types of cancer. ACP-CapsPred integrates protein language models to extract residue embeddings and incorporates peptides’ physicochemical properties and evolutionary features to construct a comprehensive profile of peptides. Employing a next-generation neural network, capsule networks, ACP-CapsPred demonstrates state-of-the-art performance in both stages. ACP-CapsPred’s convincing performance in predicting ACPs and their targets presents a novel option to expedite the development of ACPs. This study demonstrates the potential of capsule networks in protein sequence analysis. In future research, we encourage researchers to apply our proposed model architecture to other protein sequence analysis tasks, such as predicting other functional activities of proteins. Additionally, we encourage researchers to make further modifications to our model architecture, such as trying different feature encoding methods or using other protein language models to extract residue embeddings, to achieve better prediction performance.

In conclusion, ACP-CapsPred is a valuable tool for scientists studying ACPs. ACP-CapsPred significantly accelerates the screening process for ACPs, efficiently identifying ACPs from natural or synthetic peptide collections without extensive manual labor. This substantially reduces the time and effort required by researchers for screening potential ACPs, thereby lowering laboratory workload. By enabling high-throughput screening, ACP-CapsPred can help pharmaceutical companies reduce drug development costs. Furthermore, ACP-CapsPred offers better interpretability compared with other existing computational tools, revealing the importance of residues at different positions on anticancer activity, which helps researchers better understand the mechanisms of action of ACP. Clinically, physicians and scientists can use ACP-CapsPred to further optimize peptide structures through molecular docking or molecular dynamics simulations and conduct cell experiments to evaluate their toxicity and efficacy. Additionally, ACPs identified by ACP-CapsPred can be combined with existing treatments including chemotherapy, radiotherapy, and immunotherapy to form combination therapy regimens. By attacking cancer cells through multiple pathways, comprehensive treatment efficacy can be enhanced, potentially setting a new trend in cancer therapy.

**Key Points**

- This study introduces a computational framework named ACP-CapsPred, designed to identify ACPs and characterize their functional activities across various types of cancer.
- ACP-CapsPred integrates residue embeddings from a protein language model with their evolutionary information and physicochemical properties to construct a comprehensive peptide profile. ACP-CapsPred employs a next-generation neural network, specifically capsule networks, to construct classification models to enhance predictive capabilities.
- ACP-CapsPred demonstrates satisfactory predictive capabilities in both stages, achieving state-of-the-art performance.
- ACP-CapsPred exhibits excellent interpretability, revealing regions and residues associated with anticancer activity, aiding researchers in understanding the mechanism of ACP’s anticancer effects.

## Supplementary Material

supplementary_bbae460

## Data Availability

The codes and datasets of ACP-CapsPred are available at https://github.com/Cpillar/ACP-CapsPred.
