# Supplementary material for: ACP-CapsPred: an explainable computational framework for identification and functional prediction of anticancer peptides based on capsule network
Source: Brief Bioinform. 2024 Sep 18;25(5):bbae460. doi: 10.1093/bib/bbae460 (PMC11410379; doi:10.1093/bib/bbae460)
Supplement: supplementary_bbae460 [file supplementary_bbae460.pdf]

Algorithm 1: Dynamic Routing

Input: $\hat{\boldsymbol{u}}_{\boldsymbol{j}|\boldsymbol{i}}$ and $\boldsymbol{r}$

Output: $\boldsymbol{v_j}$

1. For primary capsule $\boldsymbol{i}$ and type capsule $\boldsymbol{j}$: $\boldsymbol{b_{ij}} \leftarrow \boldsymbol{0}$
2. **for** $\boldsymbol{r}$ iterations **do**:
3. $\quad \boldsymbol{c_{ij}} \leftarrow \boldsymbol{softmax}(\boldsymbol{b_{ij}})$;
4. $\quad \boldsymbol{S_j} \leftarrow \sum_i \boldsymbol{c_{ij}} \hat{\boldsymbol{u}}_{\boldsymbol{j}|\boldsymbol{i}}$;
5. $\quad \boldsymbol{v_j} \leftarrow \boldsymbol{Squash}(\boldsymbol{S_j})$;
6. $\quad \boldsymbol{b_{ij}} \leftarrow \boldsymbol{b_{ij}} + \hat{\boldsymbol{u}}_{\boldsymbol{j}|\boldsymbol{i}} \boldsymbol{v_j}$;
7. **end**
8. return $\boldsymbol{v_j}$

The algorithmic representation of dynamic routing is delineated in **Algorithm 1**. The scalar b_{ij} functions as the prior probability between the primary capsule i and the type capsule j, and c_{ij} is calculated through a softmax function applied to b_{ij}. The sum of the coupling coefficients from primary capsule i to the type capsules, $\sum_{k=1}^{N} c_{ik}$, is equal to 1, where N represents the number of type capsules. The hyperparameter r determines the number of iterations and is pre-defined. During dynamic routing, the type capsule layer generates output vectors v_j, where the elements within v_j encode features, and their magnitude signifies the probability distribution between the two types, ACP, and non-ACP.

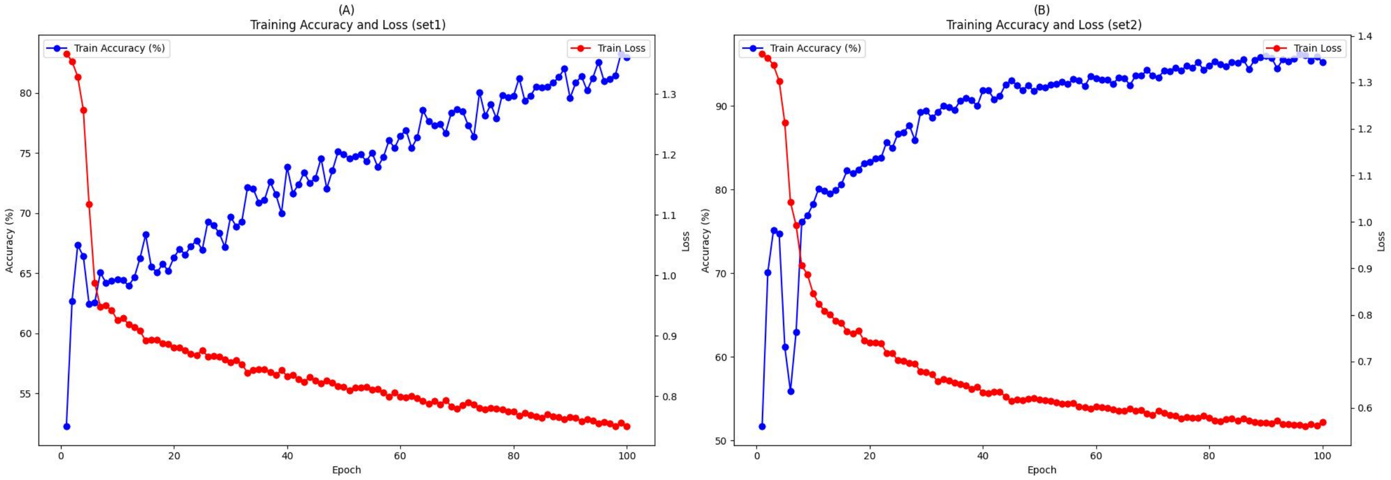

Figure S1. Training curves of ACP-CapsPred on Set 1 and Set 2.

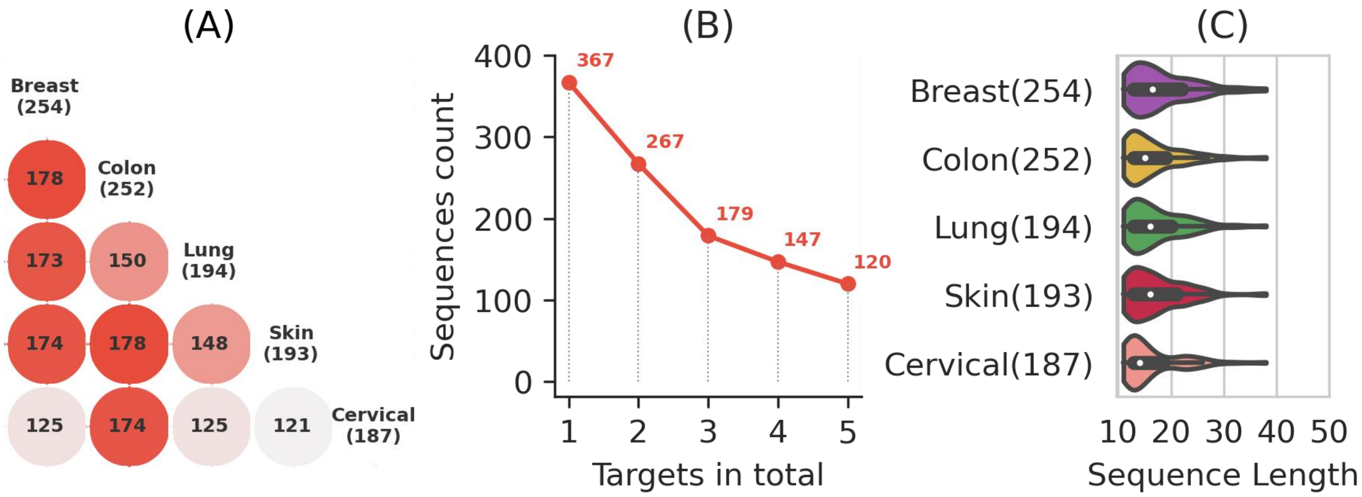

Figure S2. Statistics related to the ACP collection in the second stage. (A) A cross-counted matrix for dual-functional sequences, where each off-diagonal element denotes the count of peptide sequences concurrently exhibiting two functional activities in accordance with their diagonal target labels. (B) The quantity of ACPs based on their total surveyed targets. (C) Length distributions of ACPs categorized by their functional activity targets across different cancers.

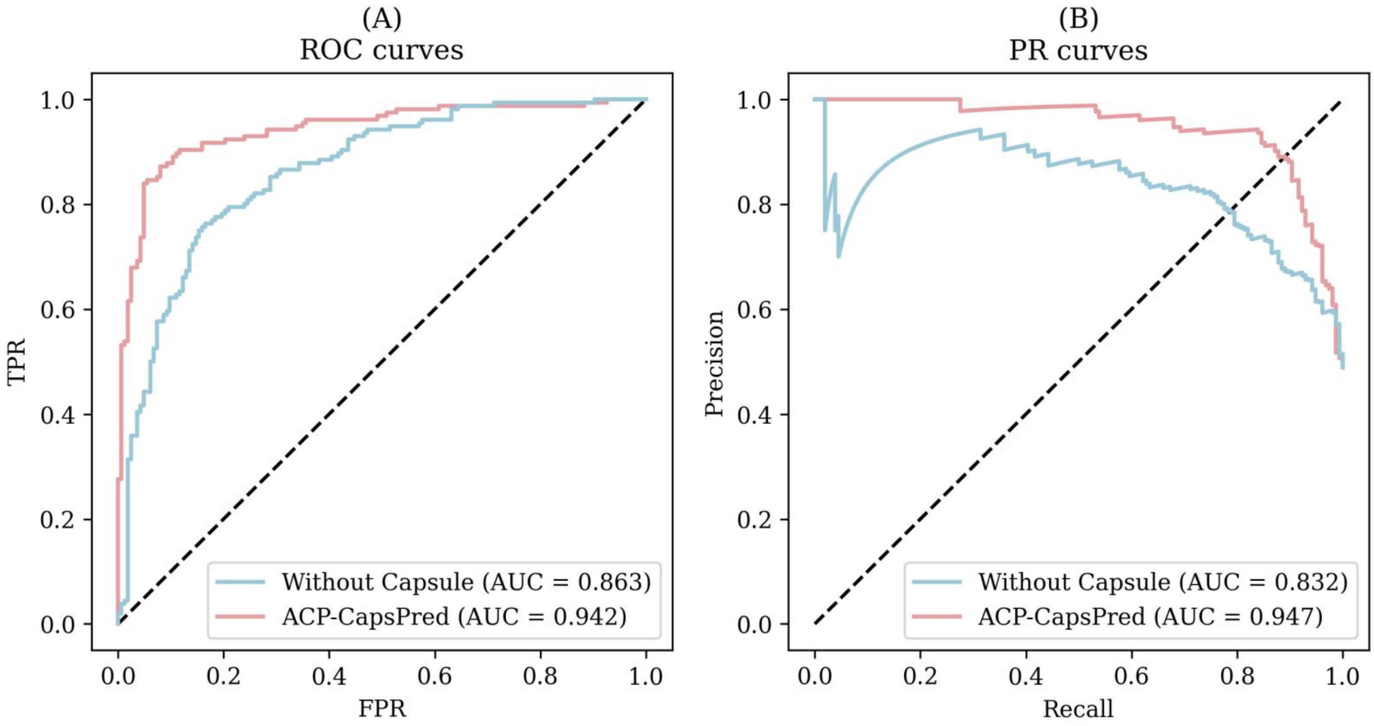

Figure S3. ROC and PR curves for ACP-CapsPred and the model without a capsule network on Set 1.

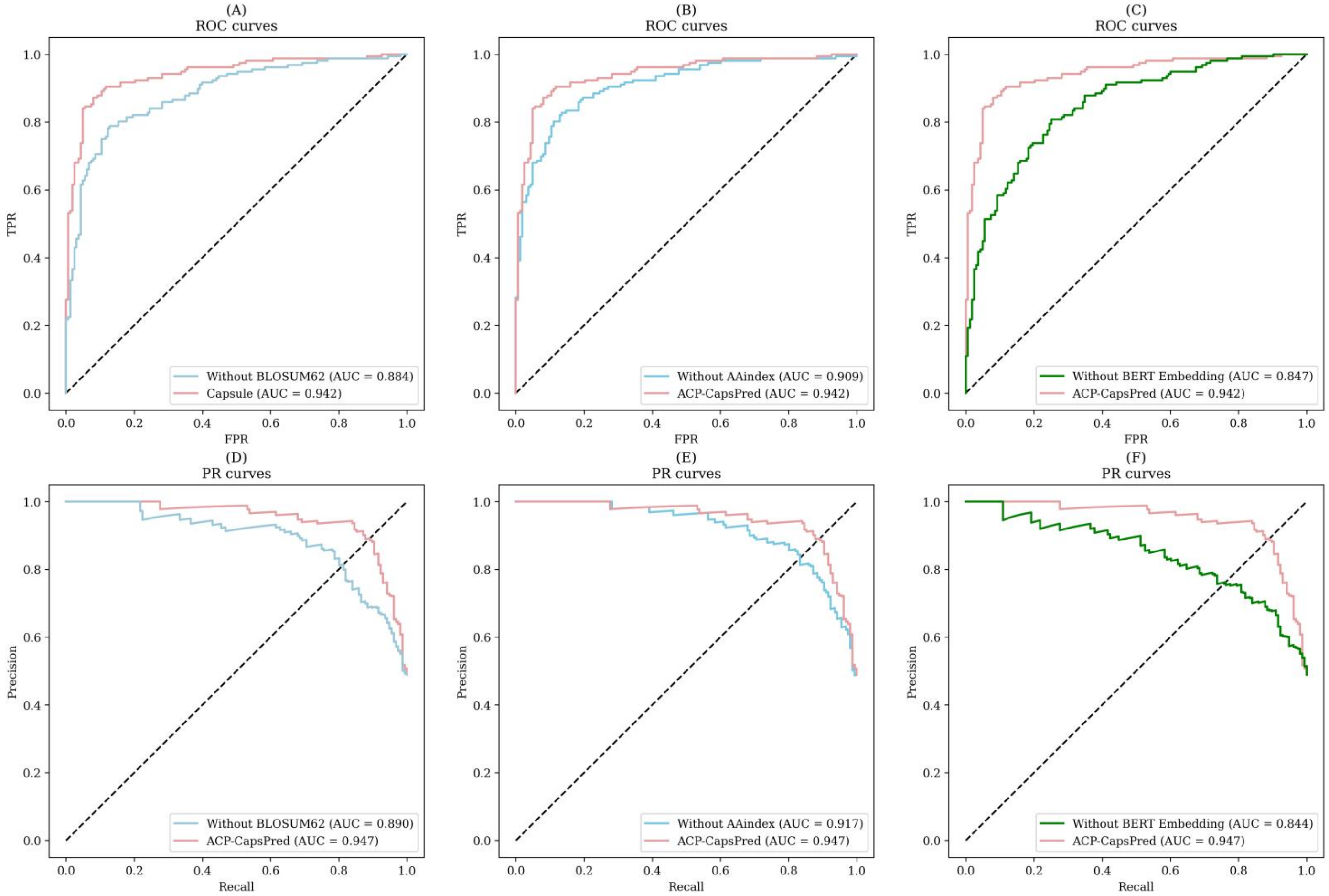

Figure S4. ROC and PR curves of the ablation experiments on Set 1.

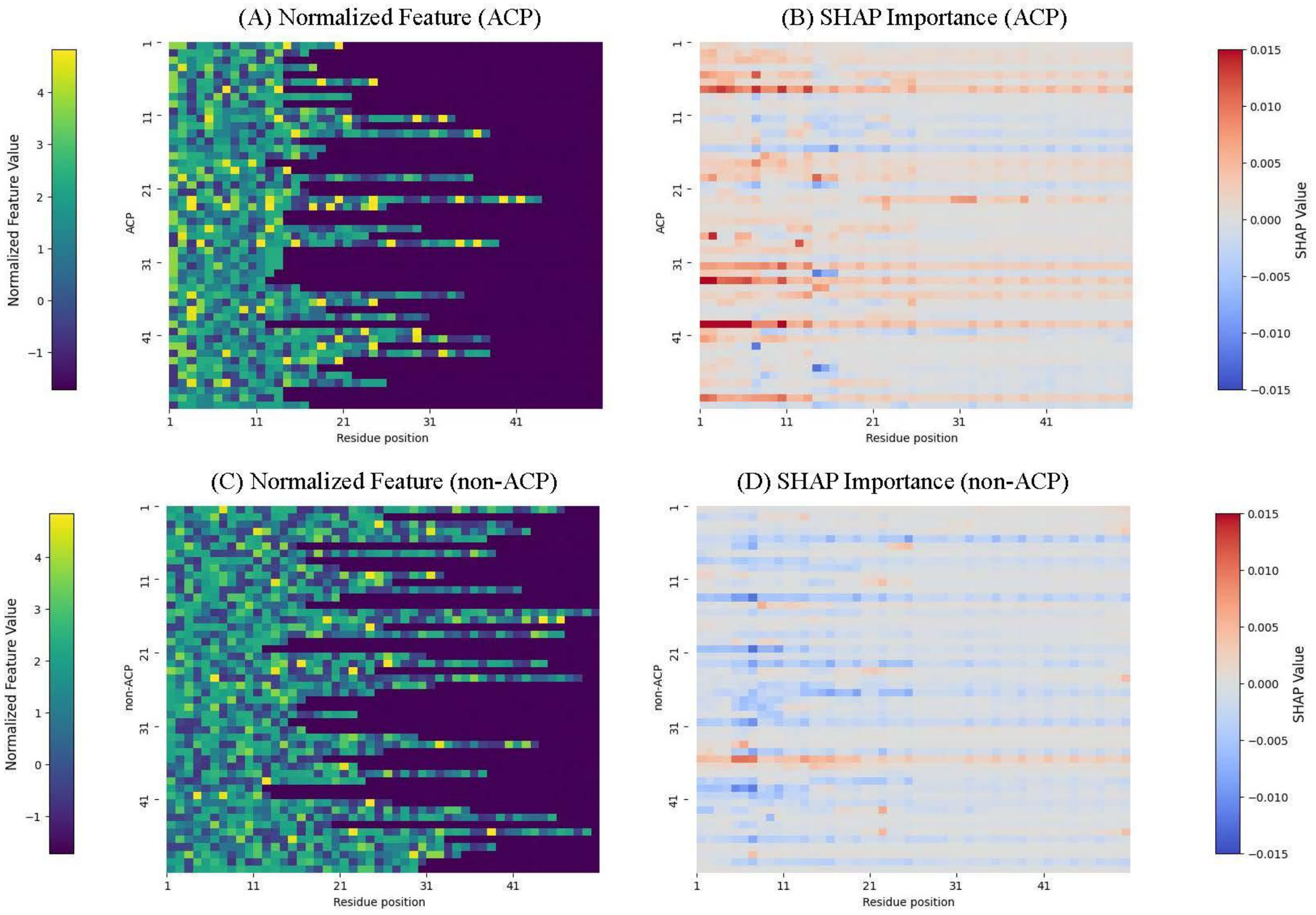

Figure S5. SHAP Analysis in this study. (A) Normalized features of different residue positions in 50 randomly selected ACPs. (B) SHAP importance of different residue positions in 50 randomly selected ACPs. (C) Normalized features of different residue positions in 50 randomly selected non-ACPs. (D) SHAP importance of different residue positions in 50 randomly selected non-ACPs.

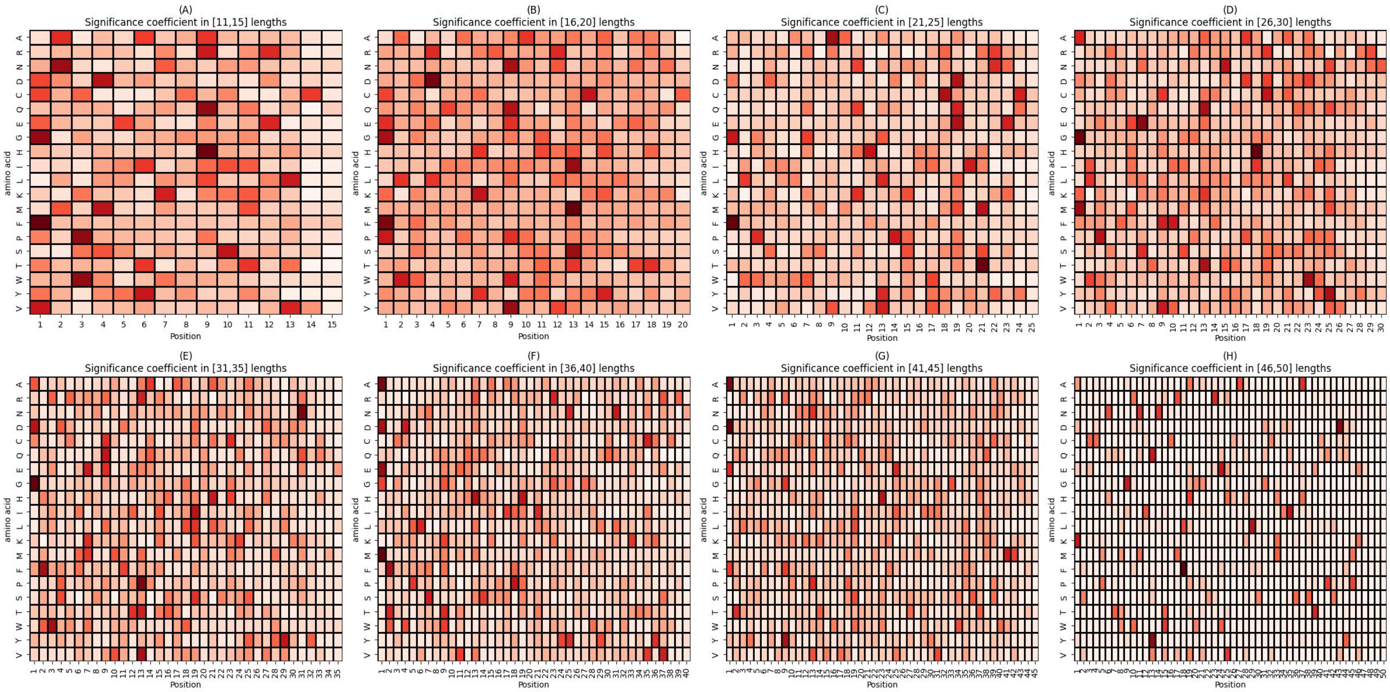

Figure S6. Heatmap of the importance of amino acid types and positions.

Table S1. Overview of datasets used in functional activity prediction of ACP against various cancers.

Cancer type	Total	Train set	Test set
Breast	254	203	51
Colon	252	202	50
Lung	194	155	39
Skin	193	154	39
Cervical	187	150	37

Table S2. The hyperparameters of ACP-CapsPred.

Hyperparameters	Search space	Best hyperparameter (Set 1)	Best hyperparameter (Set 2)
The dimension of primary capsules	{64,128,256}	256	256
The number of iterations	{2,3,4,5}	3	2
The dimension of type capsules	{4,8,16,32,64}	32	32

Table S3. Confusion matrices of ACP-CapsPred and other tools in the first stage.

Dataset	Method	TP	TN	FP	FN
Set 1 ACP versus non-ACP (AMP)	AntiCP	154	16	142	7
	AntiCP2	100	126	32	61
	AMPFun	116	103	55	45
	dbAMP	132	83	75	29
	ACPred	140	35	123	21
	iACP-GE	125	117	41	36
	StackACPred	111	122	36	50
	DeepACP	147	38	120	14
	ACP-MHCNN	152	30	128	9
	GRDF	126	120	38	35
	ACP-MLC	134	101	57	27
	cACP	101	120	38	60
	cACP-2LFS	107	109	49	54
	cACP-DeepGram	109	81	77	52
	iACP-GAEnsC	107	112	46	54
	ANNprob-ACPs	118	116	42	43
	ACP-ML	122	119	39	39
	CAPTURE	122	119	39	39
	This study	125	131	28	35
Set 2 ACP versus non-ACP (Random Peptide)	AntiCP	143	170	0	43
	AntiCP2	170	156	14	16
	AMPFun	119	156	14	67
	dbAMP	106	71	99	80
	ACPred	164	151	19	22
	iACP-GE	162	157	13	24
	StackACPred	168	163	7	18
	DeepACP	164	159	11	22
	ACP-MHCNN	164	162	8	22
	GRDF	169	166	4	17
	ACP-MLC	150	152	18	36
	cACP	166	145	25	20
	cACP-2LFS	166	130	40	20
	cACP-DeepGram	158	151	19	28
	iACP-GAEnsC	132	156	14	54
	ANNprob-ACPs	171	155	15	15
	ACP-ML	171	155	15	15
	CAPTURE	172	139	31	14
	This study	177	163	7	9

Table S4. Confusion matrices of ACP-CapsPred and ACP-MLC in the second stage.

Cancer type	Method	TP	TN	FP	FN
Breast	ACP-MLC	47	20	3	4
	ACP-CapsPred	49	21	2	3
Lung	ACP-MLC	38	5	30	0
	ACP-CapsPred	32	31	4	7
Colon	ACP-MLC	51	50	6	1
	ACP-CapsPred	47	51	5	4
Cervical	ACP-MLC	35	33	3	3
	ACP-CapsPred	36	35	1	2
Skin	ACP-MLC	29	20	14	5
	ACP-CapsPred	36	31	4	3

Table S5. Performance comparison of ACP-CapsPred and three machine learning baseline models in the second stage.

Cancer type	Method	Accuracy	Precision	Recall (=TPR)	F1-score	FPR	AUROC	AUPRC
Breast	RF	76.06%	81.82%	80.00%	80.90%	30.77%	0.785	0.883
	SVM	70.42%	68.18%	**100.00%**	81.08%	80.77%	0.821	0.843
	KNN	81.69%	77.59%	**100.00%**	87.38%	50.00%	0.886	**0.929**
	**ACP-CapsPred**	**94.59%**	**96.08%**	96.08%	**96.08%**	**3.92%**	**0.905**	0.885
Lung	RF	77.46%	76.32%	80.56%	78.38%	25.71%	0.774	0.834
	SVM	77.46%	**91.67%**	61.11%	73.33%	**5.71%**	0.848	0.851
	KNN	78.87%	70.59%	**100.00%**	82.76%	42.86%	0.836	0.850
	**ACP-CapsPred**	**82.43%**	85.14%	82.05%	**83.57%**	11.42%	**0.856**	**0.854**
Colon	RF	77.46%	79.66%	92.16%	85.45%	60.00%	0.767	0.907
	SVM	80.28%	**92.31%**	66.67%	77.42%	**5.71%**	0.906	0.923
	KNN	83.10%	84.21%	**94.12%**	88.89%	45.00%	0.853	0.942
	**ACP-CapsPred**	**91.60%**	90.83%	92.16%	**91.49%**	8.92%	**0.910**	**0.959**
Cervical	RF	81.69%	80.95%	87.18%	83.95%	25.00%	0.932	0.948
	SVM	83.10%	86.49%	82.05%	84.21%	15.63%	0.915	0.932
	KNN	84.51%	83.33%	89.74%	86.42%	21.88%	0.910	0.926
	**ACP-CapsPred**	**94.59%**	**92.11%**	**97.22%**	**94.60%**	**2.77%**	**0.950**	**0.970**
Skin	RF	81.69%	83.33%	85.37%	84.34%	23.33%	0.810	0.886
	SVM	85.92%	**96.97%**	78.05%	86.49%	**3.33%**	0.922	0.938
	KNN	85.92%	82.98%	**95.12%**	88.64%	26.67%	0.891	0.923
	**ACP-CapsPred**	**90.54%**	90.00%	92.31%	**91.14%**	11.42%	**0.968**	**0.974**
Average	RF	78.87%	80.42%	85.05%	82.60%	32.96%	0.814	0.892
	SVM	79.44%	87.12%	77.58%	80.51%	22.23%	0.882	0.897
	KNN	82.82%	79.74%	**95.80%**	86.82%	37.28%	0.875	0.914
	**ACP-CapsPred**	**90.75%**	**90.83%**	91.96%	**91.38%**	**7.69%**	**0.918**	**0.928**

Note: Three machine learning baselines employ five common peptide descriptors, including AAC, DPC, PAAC, CKSAAGP, and PHYC.

Table S6. Performance comparison of different dimensionality reduction methods on the two datasets.

Dataset	Dimensionality Reduction Method	Accuracy	Precision	Recall (=TPR)	F1-score	FPR	AUROC	AUPRC
Set 1	Factor Analysis	75.23%	78.94%	67.31%	72.66%	22.22%	0.825	0.835
	Independent Component Analysis	70.22%	70.75%	66.67%	68.64%	26.38%	0.754	0.738
	UMAP	70.53%	69.87%	69.87%	65.45%	17.79%	0.784	0.798
	**This study (PCA)**	**80.25%**	**81.70%**	**78.12%**	**79.86%**	**17.61%**	**0.942**	**0.947**
Set 2	Factor Analysis	93.14%	93.60%	92.52%	93.06%	6.25%	0.974	0.981
	Independent Component Analysis	82.86%	81.66%	84.48%	83.05%	18.75%	0.898	0.917
	UMAP	85.42%	89.67%	79.89%	84.49%	9.09%	0.909	0.934
	**This study (PCA)**	**95.71%**	**95.98%**	**95.43%**	**95.90%**	**4.12%**	**0.986**	**0.989**

Table S7. Performance comparison between the encoding method used in ACP-CapsPred and one-hot encoding.

Dataset	Encoding Method	Accuracy	Precision	Recall (=TPR)	F1-score	FPR	AUROC	AUPRC
Set 1	One-hot encoding	72.73%	76.09%	66.03%	70.71%	20.62%	0.774	0.797
	**This study**	**80.25%**	**81.70%**	**78.12%**	**79.86%**	**17.61%**	**0.942**	**0.947**
Set 2	One-hot encoding	89.71%	91.81%	86.21%	88.89%	8.09%	0.941	0.952
	**This study**	**95.71%**	**95.98%**	**95.43%**	**95.90%**	**4.12%**	**0.986**	**0.989**

Physicochemical properties of AAindex involved in this study

No.	AAIndex	Description
1	LEVM760107	van der Waals parameter epsilon (Levitt, 1976)
2	CHAM820101	Polarizability parameter (Charton-Charton, 1982)
3	FASG760101	Molecular weight (Fasman, 1976)
4	OOBM770104	Average non-bonded energy per residue (Oobatake-Ooi, 1977)
5	HARY940101	Partition energy (Guy, 1985)
6	PONJ960101	Partition coefficient (Pliska et al., 1981)
7	GARJ730101	Partition coefficient (Garel et al., 1973)
8	CHOC760101	Residue accessible surface area in tripeptide (Chothia, 1976)
9	FAUJ880106	STERIMOL maximum width of the side chain (Fauchere et al., 1988)
10	RACS820107	Average relative fractional occurrence in A0(i-1) (Rackovsky-Scheraga,
11	ROSG850101	Mean area buried on transfer (Rose et al., 1985)
12	TSAJ990102	Normalized frequency of chain reversal (Tanaka-Scheraga, 1977)
13	NISK800101	8 A contact number (Nishikawa-Ooi, 1980)
14	GOLD730102	Residue volume (Goldsack-Chalifoux, 1973)
15	GRAR740103	Volume (Grantham, 1974)
16	MEIH800103	Average side chain orientation angle (Meirovitch et al., 1980)
17	RICJ880104	Relative preference value at N1 (Richardson-Richardson, 1988)
18	WEBA780101	RF value in high salt chromatography (Weber-Lacey, 1978)
19	CHOC750101	Average volume of buried residue (Chothia, 1975)
20	BIGC670101	Residue volume (Bigelow, 1967)
21	DESM900101	Membrane preference for cytochrome b: MPH89 (Degli Esposti et al., 1990
22	FAUJ880103	Normalized van der Waals volume (Fauchere et al., 1988)
23	LEVM760105	Radius of gyration of side chain (Levitt, 1976)
24	PARS000102	HPLC parameter (Parker et al., 1986)
25	ZASB820101	Linker propensity from non-helical (annotated by DSSP) dataset (George
26	MCMT640101	Refractivity (McMeekin et al., 1964), Cited by Jones (1975)
27	CHAM830106	The number of bonds in the longest chain (Charton-Charton, 1983)
28	TAKK010101	Optimal matching hydrophobicity (Sweet-Eisenberg, 1983)
29	OOBM770103	Long range non-bonded energy per atom (Oobatake-Ooi, 1977)
30	PONP800108	Average number of surrounding residues (Ponnuswamy et al., 1980)
31	CIDH920102	Normalized hydrophobicity scales for beta-proteins (Cid et al., 1992)
32	VASM830103	Relative population of conformational state E (Vasquez et al., 1983)
33	JOND750101	Hydrophobicity (Jones, 1975)
34	LIFS790101	Conformational preference for all beta-strands (Lifson-Sander, 1979)
35	AURR980105	Membrane-buried preference parameters (Argos et al., 1982)
36	WOLS870103	Linker propensity from non-helical (annotated by DSSP) dataset (George
37	WIMW960101	Linker propensity from non-helical (annotated by DSSP) dataset (George
38	KIMC930101	The Kerr-constant increments (Khanarian-Moore, 1980)
39	KARP850101	Flexibility parameter for no rigid neighbors (Karplus-Schulz, 1985)
40	KRIW790101	Side chain interaction parameter (Krigbaum-Komoriya, 1979)
41	CIDH920105	Normalized average hydrophobicity scales (Cid et al., 1992)
42	VASM830102	Relative population of conformational state C (Vasquez et al., 1983)
43	CORJ870101	Partial specific volume (Cohn-Edsall, 1943)
44	ARGP820101	Hydrophobicity index (Argos et al., 1982)
45	EISD860101	Solvation free energy (Eisenberg-McLachlan, 1986)
46	GRAR740101	Composition (Grantham, 1974)
47	FUKS010104	Helix termination parameter at posision j+1 (Finkelstein et al., 1991)

48	OOBM850102	Optimized propensity to form reverse turn (Oobatake et al., 1985)
49	SIMZ760101	Transfer free energy (Simon, 1976), Cited by Charton-Charton (1982)
50	SNEP660103	Principal component III (Sneath, 1966)
51	TSAJ990101	Normalized frequency of chain reversal (Tanaka-Scheraga, 1977)
52	MEEJ810102	Retention coefficient in NaH2PO4 (Meek-Rossetti, 1981)
53	OOBM850104	Optimized average non-bonded energy per atom (Oobatake et al., 1985)
54	OOBM770102	Short and medium range non-bonded energy per atom (Oobatake-Ooi, 1977)
55	LEVM760102	Distance between C-alpha and centroid of side chain (Levitt, 1976)
56	RICJ880111	Relative preference value at C4 (Richardson-Richardson, 1988)
57	CORJ870103	Partial specific volume (Cohn-Edsall, 1943)
58	KRIW790103	Side chain volume (Krigbaum-Komoriya, 1979)
59	JANJ790101	Ratio of buried and accessible molar fractions (Janin, 1979)
60	OOBM770105	Short and medium range non-bonded energy per residue (Oobatake-Ooi, 1979)
61	NADH010107	Effective partition energy (Miyazawa-Jernigan, 1985)
62	MANP780101	Average surrounding hydrophobicity (Manavalan-Ponnuswamy, 1978)
63	GEIM800105	Beta-strand indices (Geisow-Roberts, 1980)
64	LIFS790103	Conformational preference for antiparallel beta-strands (Lifson-Sander,
65	PONP800106	Surrounding hydrophobicity in turn (Ponnuswamy et al., 1980)
66	ZHOH040102	The relative stability scale extracted from mutation experiments (Zhou-
67	QIAN880130	Weights for coil at the window position of -3 (Qian-Sejnowski, 1988)
68	ZHOH040101	The stability scale from the knowledge-based atom-atom potential (Zhou-
69	PONP800102	Average gain in surrounding hydrophobicity (Ponnuswamy et al., 1980)
70	CHOP780205	Normalized frequency of C-terminal helix (Chou-Fasman, 1978b)
71	VINM940104	Transfer free energy to lipophilic phase (von Heijne-Blomberg, 1979)
72	FODM020101	Helix termination parameter at posision j+1 (Finkelstein et al., 1991)
73	CHAM830104	The number of atoms in the side chain labelled 2+1 (Charton-Charton, 19
74	DESM900102	Average membrane preference: AMP07 (Degli Esposti et al., 1990)
75	GEOR030108	Aperiodic indices for alpha/beta-proteins (Geisow-Roberts, 1980)
76	MIYS990105	Effective partition energy (Miyazawa-Jernigan, 1985)
77	FAUJ880109	Number of hydrogen bond donors (Fauchere et al., 1988)
78	BEGF750103	Conformational parameter of beta-turn (Beghin-Dirkx, 1975)
79	AURR980104	Membrane-buried preference parameters (Argos et al., 1982)
80	FINA910104	Helix termination parameter at posision j+1 (Finkelstein et al., 1991)
81	RACS820112	Average relative fractional occurrence in ER(i-1) (Rackovsky-Scheraga,
82	DAWD720101	Size (Dawson, 1972)
83	ROBB790101	Hydration free energy (Robson-Osguthorpe, 1979)
84	LEVM760101	Hydrophobic parameter (Levitt, 1976)
85	QIAN880139	Weights for coil at the window position of 6 (Qian-Sejnowski, 1988)
86	RADA880106	Accessible surface area (Radzicka-Wolfenden, 1988)
87	EISD840101	Consensus normalized hydrophobicity scale (Eisenberg, 1984)
88	KANM800102	Average relative probability of beta-sheet (Kanehisa-Tsong, 1980)
89	KARP850103	Flexibility parameter for two rigid neighbors (Karplus-Schulz, 1985)
90	YUTK870103	Linker propensity from non-helical (annotated by DSSP) dataset (George
91	BROC820101	Retention coefficient in TFA (Browne et al., 1982)
92	QIAN880126	Weights for beta-sheet at the window position of 6 (Qian-Sejnowski, 198
93	KHAG800101	The Kerr-constant increments (Khanarian-Moore, 1980)
94	GEIM800108	Aperiodic indices (Geisow-Roberts, 1980)
95	NADH010103	Effective partition energy (Miyazawa-Jernigan, 1985)
96	FASG760102	Melting point (Fasman, 1976)

97	JANJ780101	Average accessible surface area (Janin et al., 1978)
98	WERD780104	Linker propensity from non-helical (annotated by DSSP) dataset (George
99	FASG890101	pK-C (Fasman, 1976
100	ROSG850102	Mean fractional area loss (Rose et al., 1985)
101	NOZY710101	Transfer energy, organic solvent/water (Nozaki-Tanford, 1971)
102	AURR980101	Membrane-buried preference parameters (Argos et al., 1982)
103	CHOP780210	Normalized frequency of N-terminal non beta region (Chou-Fasman, 1978b)
104	VENT840101	Bitterness (Venanzi, 1984)
105	FUKS010102	Helix termination parameter at posision j+1 (Finkelstein et al., 1991)
106	NADH010106	Effective partition energy (Miyazawa-Jernigan, 1985)
107	LEVM780101	Normalized frequency of alpha-helix, with weights (Levitt, 1978)
108	MEEJ800102	Retention coefficient in HPLC, pH2.1 (Meek, 1980)
109	PONP800105	Surrounding hydrophobicity in beta-sheet (Ponnuswamy et al., 1980)
110	PRAM900102	Relative frequency in alpha-helix (Prabhakaran, 1990)
111	PALJ810104	Normalized frequency of beta-sheet from CF (Palau et al., 1981)
112	AURR980114	Membrane-buried preference parameters (Argos et al., 1982)
113	CORJ870107	Partial specific volume (Cohn-Edsall, 1943)
114	CHOP780202	Normalized frequency of beta-sheet (Chou-Fasman, 1978b)
115	MEEJ810101	Retention coefficient in NaClO4 (Meek-Rossetti, 1981)
116	RACS770101	Average reduced distance for C-alpha (Rackovsky-Scheraga, 1977)
117	VINM940101	Transfer free energy to lipophilic phase (von Heijne-Blomberg, 1979)
118	PLIV810101	Partition coefficient (Pliska et al., 1981)
119	RICJ880105	Relative preference value at N2 (Richardson-Richardson, 1988)
120	KARP850102	Flexibility parameter for one rigid neighbor (Karplus-Schulz, 1985)
121	PRAM820103	Correlation coefficient in regression analysis (Prabhakaran-Ponnuswamy,
122	FAUJ880104	STERIMOL length of the side chain (Fauchere et al., 1988)
123	GOLD730101	Hydrophobicity factor (Goldsack-Chalifoux, 1973)
124	PRAM900104	Relative frequency in reverse-turn (Prabhakaran, 1990)
125	KIDA850101	The Kerr-constant increments (Khanarian-Moore, 1980)
126	YUTK870102	Linker propensity from non-helical (annotated by DSSP) dataset (George
127	MEEJ800101	Retention coefficient in HPLC, pH7.4 (Meek, 1980)
128	PALJ810111	Normalized frequency of beta-sheet in alpha+beta class (Palau et al., 1
129	AURR980119	Membrane-buried preference parameters (Argos et al., 1982)
130	FUKS010108	Helix termination parameter at posision j+1 (Finkelstein et al., 1991)
131	RADA880107	Energy transfer from out to in(95%buried) (Radzicka-Wolfenden, 1988)
132	RICJ880116	Relative preference value at C' (Richardson-Richardson, 1988)
133	FAUJ880101	Graph shape index (Fauchere et al., 1988)
134	LEWP710101	Frequency of occurrence in beta-bends (Lewis et al., 1971)
135	RADA880103	Transfer free energy from vap to chx (Radzicka-Wolfenden, 1988)
136	PALJ810116	Normalized frequency of turn in alpha/beta class (Palau et al., 1981)
137	PONP800101	Surrounding hydrophobicity in folded form (Ponnuswamy et al., 1980)
138	WOLS870101	Linker propensity from non-helical (annotated by DSSP) dataset (George
139	SUYM030101	Zimm-Bragg parameter sigma x 1.0E4 (Sueki et al., 1984)
140	BAEK050101	Membrane-buried preference parameters (Argos et al., 1982)
141	CORJ870108	Partial specific volume (Cohn-Edsall, 1943)
142	WILM950102	Linker propensity from non-helical (annotated by DSSP) dataset (George
143	CORJ870104	Partial specific volume (Cohn-Edsall, 1943)
144	CIDH920101	Normalized hydrophobicity scales for alpha-proteins (Cid et al., 1992)
145	PALJ810112	Normalized frequency of beta-sheet in alpha/beta class (Palau et al., 1

146	QIAN880118	Weights for beta-sheet at the window position of -2 (Qian-Sejnowski, 19
147	SUEM840101	Zimm-Bragg parameter s at 20 C (Sueki et al., 1984)
148	BHAR880101	Average flexibility indices (Bhaskaran-Ponnuswamy, 1988)
149	BROC820102	Retention coefficient in HFBA (Browne et al., 1982)
150	PONP800103	Average gain ratio in surrounding hydrophobicity (Ponnuswamy et al., 19
151	RACS770103	Side chain orientational preference (Rackovsky-Scheraga, 1977)
152	SNEP660104	Principal component IV (Sneath, 1966)
153	CHOP780211	Normalized frequency of C-terminal non beta region (Chou-Fasman, 1978b)
154	HOPT810101	Hydrophilicity value (Hopp-Woods, 1981)
155	ISOY800103	Normalized relative frequency of bend (Isogai et al., 1980)
156	LEVM780103	Normalized frequency of reverse turn, with weights (Levitt, 1978)
157	BUNA790103	Spin-spin coupling constants 3JHalpha-NH (Bundi-Wuthrich, 1979)
158	RACS820105	Average relative fractional occurrence in E0(i) (Rackovsky-Scheraga, 19
159	HOPA770101	Hydration number (Hopfinger, 1971), Cited by Charton-Charton (1982)
160	PALJ810115	Normalized frequency of turn in alpha+beta class (Palau et al., 1981)
161	NADH010104	Effective partition energy (Miyazawa-Jernigan, 1985)
162	ENGD860101	Direction of hydrophobic moment (Eisenberg-McLachlan, 1986)
163	LEVM780106	Normalized frequency of reverse turn, unweighted (Levitt, 1978)
164	RACS820104	Average relative fractional occurrence in EL(i) (Rackovsky-Scheraga, 19
165	CORJ870102	Partial specific volume (Cohn-Edsall, 1943)
166	KUHL950101	Side chain volume (Krigbaum-Komoriya, 1979)
167	JOND920102	Relative mutability (Jones et al., 1992)
168	NADH010105	Effective partition energy (Miyazawa-Jernigan, 1985)
169	CHOP780207	Normalized frequency of C-terminal non helical region (Chou-Fasman, 197
170	OOBM770101	Average non-bonded energy per atom (Oobatake-Ooi, 1977)
171	RICJ880109	Relative preference value at Mid (Richardson-Richardson, 1988)
172	GUYH850105	Partition energy (Guy, 1985)
173	LEVM760106	van der Waals parameter R0 (Levitt, 1976)
174	NAKH900105	AA composition of mt-proteins from animal (Nakashima et al., 1990)
175	RICJ880112	Relative preference value at C3 (Richardson-Richardson, 1988)
176	WOEC730101	Linker propensity from non-helical (annotated by DSSP) dataset (George
177	WILM950101	Linker propensity from non-helical (annotated by DSSP) dataset (George
178	GUOD860101	Volume (Grantham, 1974)
179	DIGM050101	Average membrane preference: AMP07 (Degli Esposti et al., 1990)
180	GEIM800103	Alpha-helix indices for beta-proteins (Geisow-Roberts, 1980)
181	OOBM850101	Optimized beta-structure-coil equilibrium constant (Oobatake et al., 19
182	QIAN880110	Weights for alpha-helix at the window position of 3 (Qian-Sejnowski, 19
183	WOLR810101	Linker propensity from non-helical (annotated by DSSP) dataset (George
184	CHOP780203	Normalized frequency of beta-turn (Chou-Fasman, 1978b)
185	PALJ810103	Normalized frequency of beta-sheet from LG (Palau et al., 1981)
186	QIAN880131	Weights for coil at the window position of -2 (Qian-Sejnowski, 1988)
187	RICJ880103	Relative preference value at N-cap (Richardson-Richardson, 1988)
188	CASG920101	Normalized frequency of extended structure (Burgess et al., 1974)
189	KANM800101	Average relative probability of helix (Kanehisa-Tsong, 1980)
190	PRAM900101	Hydrophobicity (Prabhakaran, 1990)
191	ROSM880101	Side chain hydropathy, uncorrected for solvation (Roseman, 1988)
192	AURR980102	Membrane-buried preference parameters (Argos et al., 1982)
193	MIYS990104	Effective partition energy (Miyazawa-Jernigan, 1985)
194	BIOV880101	Information value for accessibility; average fraction 35% (Biou et al.,

195	BUNA790101	alpha-NH chemical shifts (Bundi-Wuthrich, 1979)
196	HUTJ700102	Absolute entropy (Hutchens, 1970)
197	MIYS850101	Effective partition energy (Miyazawa-Jernigan, 1985)
198	BEGF750102	Conformational parameter of beta-structure (Beghin-Dirkx, 1975)
199	GEIM800107	Beta-strand indices for alpha/beta-proteins (Geisow-Roberts, 1980)
200	QIAN880125	Weights for beta-sheet at the window position of 5 (Qian-Sejnowski, 198
201	WOLS870102	Linker propensity from non-helical (annotated by DSSP) dataset (George
202	ZIMJ680102	Weighted second smallest eigenvalue of the weighted Laplacian matrix (K
203	VINM940102	Transfer free energy to lipophilic phase (von Heijne-Blomberg, 1979)
204	ISOY800102	Normalized relative frequency of extended structure (Isogai et al., 198
205	PARJ860101	HPLC parameter (Parker et al., 1986)
206	SUEM840102	Zimm-Bragg parameter sigma x 1.0E4 (Sueki et al., 1984)
207	JACR890101	Normalized relative frequency of coil (Isogai et al., 1980)
208	MIYS990101	Effective partition energy (Miyazawa-Jernigan, 1985)
209	MONM990101	Effective partition energy (Miyazawa-Jernigan, 1985)
210	KYTJ820101	Hydropathy index (Kyte-Doolittle, 1982)
211	GEOR030103	Aperiodic indices for alpha/beta-proteins (Geisow-Roberts, 1980)
212	MIYS990102	Effective partition energy (Miyazawa-Jernigan, 1985)
213	TANS770104	Normalized frequency of chain reversal R (Tanaka-Scheraga, 1977)
214	FUKS010103	Helix termination parameter at posision j+1 (Finkelstein et al., 1991)
215	BASU050102	Membrane-buried preference parameters (Argos et al., 1982)
216	PUNT030101	Beta-coil equilibrium constant (Ptitsyn-Finkelstein, 1983)
217	NISK860101	14 A contact number (Nishikawa-Ooi, 1986)
218	ROBB760110	Information measure for middle turn (Robson-Suzuki, 1976)
219	KRIW710101	Side chain interaction parameter (Krigbaum-Rubin, 1971)
220	LEVM780105	Normalized frequency of beta-sheet, unweighted (Levitt, 1978)
221	RADA880101	Transfer free energy from chx to wat (Radzicka-Wolfenden, 1988)
222	RADA880105	Transfer free energy from vap to oct (Radzicka-Wolfenden, 1988)
223	MAXF760101	Normalized frequency of alpha-helix (Maxfield-Scheraga, 1976)
224	NAKH920105	AA composition of MEM of single-spanning proteins
225	PALJ810107	Normalized frequency of alpha-helix in all-alpha class (Palau et al., 1
226	PTIO830102	Beta-coil equilibrium constant (Ptitsyn-Finkelstein, 1983)
227	BASU050101	Membrane-buried preference parameters (Argos et al., 1982)
228	NADH010101	Effective partition energy (Miyazawa-Jernigan, 1985)
229	MITS020101	Average side chain orientation angle (Meirovitch et al., 1980)
230	NAKH900103	AA composition of mt-proteins (Nakashima et al., 1990)
231	QIAN880120	Weights for beta-sheet at the window position of 0 (Qian-Sejnowski, 198
232	SNEP660102	Principal component II (Sneath, 1966)
233	ZHOH040103	Buriability (Zhou-Zhou, 2004)
234	RACS820102	Average relative fractional occurrence in AR(i) (Rackovsky-Scheraga, 19
235	SWER830101	Optimal matching hydrophobicity (Sweet-Eisenberg, 1983)
236	MIYS990103	Effective partition energy (Miyazawa-Jernigan, 1985)
237	FINA770101	Helix-coil equilibrium constant (Finkelstein-Ptitsyn, 1977)
238	PRAM820101	Intercept in regression analysis (Prabhakaran-Ponnuswamy, 1982)
239	ARGP820103	Membrane-buried preference parameters (Argos et al., 1982)
240	FAUJ880110	Number of full nonbonding orbitals (Fauchere et al., 1988)
241	ANDN920101	alpha-CH chemical shifts (Andersen et al., 1992)
242	CHAM830105	The number of atoms in the side chain labelled 3+1 (Charton-Charton, 19
243	VINM940103	Transfer free energy to lipophilic phase (von Heijne-Blomberg, 1979)

244	BULH740101	Transfer free energy to surface (Bull-Breese, 1974)
245	FASG760104	pK-N (Fasman, 1976)
246	JANJ780102	Percentage of buried residues (Janin et al., 1978)
247	QIAN880122	Weights for beta-sheet at the window position of 2 (Qian-Sejnowski, 198
248	RADA880104	Transfer free energy from chx to oct (Radzicka-Wolfenden, 1988)
249	RICJ880107	Relative preference value at N4 (Richardson-Richardson, 1988)
250	RICJ880114	Relative preference value at C1 (Richardson-Richardson, 1988)
251	ROBB760106	Information measure for pleated-sheet (Robson-Suzuki, 1976)
252	PARS000101	HPLC parameter (Parker et al., 1986)
253	CHOP780209	Normalized frequency of C-terminal beta-sheet (Chou-Fasman, 1978b)
254	EISD860102	Atom-based hydrophobic moment (Eisenberg-McLachlan, 1986)
255	GEIM800106	Beta-strand indices for beta-proteins (Geisow-Roberts, 1980)
256	JANJ780103	Percentage of exposed residues (Janin et al., 1978)
257	PALJ810114	Normalized frequency of turn in all-beta class (Palau et al., 1981)
258	WERD780101	Propensity to be buried inside (Wertz-Scheraga, 1978)
259	JOND750102	pK (-COOH) (Jones, 1975)
260	KRIW790102	Fraction of site occupied by water (Krigbaum-Komoriya, 1979)
261	RADA880108	Mean polarity (Radzicka-Wolfenden, 1988)
262	WOLR790101	Linker propensity from non-helical (annotated by DSSP) dataset (George
263	CHOP780213	Frequency of the 2nd residue in turn (Chou-Fasman, 1978b)
264	GEIM800101	Alpha-helix indices (Geisow-Roberts, 1980)
265	NAKH900113	Ratio of average and computed composition (Nakashima et al., 1990)
266	SNEP660101	Principal component I (Sneath, 1966)
267	CORJ870106	Partial specific volume (Cohn-Edsall, 1943)
268	LEVM780102	Normalized frequency of beta-sheet, with weights (Levitt, 1978)
269	PRAM900103	Relative frequency in beta-sheet (Prabhakaran, 1990)
270	MUNV940104	Effective partition energy (Miyazawa-Jernigan, 1985)
271	ISOY800101	Normalized relative frequency of alpha-helix (Isogai et al., 1980)
272	QIAN880128	Weights for coil at the window position of -5 (Qian-Sejnowski, 1988)
273	KUMS000103	Side chain volume (Krigbaum-Komoriya, 1979)
274	GUYH850104	Partition energy (Guy, 1985)
275	BIOV880102	Information value for accessibility; average fraction 23% (Biou et al.,
276	PONP800104	Surrounding hydrophobicity in alpha-helix (Ponnuswamy et al., 1980)
277	CORJ870105	Partial specific volume (Cohn-Edsall, 1943)
278	ARGP820102	Signal sequence helical potential (Argos et al., 1982)
279	CHOP780216	Normalized frequency of the 2nd and 3rd residues in turn (Chou-Fasman,
280	FINA910101	Helix initiation parameter at posision i-1 (Finkelstein et al., 1991)
281	LIFS790102	Conformational preference for parallel beta-strands (Lifson-Sander, 197
282	NAKH920102	AA composition of CYT2 of single-spanning proteins (Nakashima-Nishikawa
283	VASM830101	Relative population of conformational state A (Vasquez et al., 1983)
284	ZIMJ680103	Weighted second smallest eigenvalue of the weighted Laplacian matrix (K
285	BUNA790102	alpha-CH chemical shifts (Bundi-Wuthrich, 1979)
286	RICJ880113	Relative preference value at C2 (Richardson-Richardson, 1988)
287	WERD780103	Linker propensity from non-helical (annotated by DSSP) dataset (George
288	JURD980101	Sequence frequency (Jungck, 1978)
289	JANJ790102	Transfer free energy (Janin, 1979)
290	NAKH920103	AA composition of EXT of single-spanning proteins (Nakashima-Nishikawa,
291	NAKH920108	AA composition of MEM of multi-spanning proteins (Nakashima-Nishikawa,
292	RICJ880117	Relative preference value at C" (Richardson-Richardson, 1988)

293	COWR900101	Partial specific volume (Cohn-Edsall, 1943)
294	CHOP780215	Frequency of the 4th residue in turn (Chou-Fasman, 1978b)
295	GRAR740102	Polarity (Grantham, 1974)
296	HUTJ700103	Entropy of formation (Hutchens, 1970)
297	CHOC760102	Residue accessible surface area in folded protein (Chothia, 1976)
298	CHOP780208	Normalized frequency of N-terminal beta-sheet (Chou-Fasman, 1978b)
299	OOBM850103	Optimized transfer energy parameter (Oobatake et al., 1985)
300	CEDJ970105	Normalized frequency of extended structure (Burgess et al., 1974)
301	CIDH920103	Normalized hydrophobicity scales for alpha+beta-proteins (Cid et al., 1
302	GEIM800110	Aperiodic indices for beta-proteins (Geisow-Roberts, 1980)
303	PALJ810110	Normalized frequency of beta-sheet in all-beta class (Palau et al., 198
304	CHAM830107	A parameter of charge transfer capability (Charton-Charton, 1983)
305	NADH010102	Effective partition energy (Miyazawa-Jernigan, 1985)
306	QIAN880115	Weights for beta-sheet at the window position of -5 (Qian-Sejnowski, 19
307	PONP930101	Average number of surrounding residues (Ponnuswamy et al., 1980)
308	KLEP840101	Net charge (Klein et al., 1984)
309	LEVM780104	Normalized frequency of alpha-helix, unweighted (Levitt, 1978)
310	MAXF760103	Normalized frequency of zeta R (Maxfield-Scheraga, 1976)
311	PALJ810109	Normalized frequency of alpha-helix in alpha/beta class (Palau et al.,
312	KUMS000104	Side chain volume (Krigbaum-Komoriya, 1979)
313	CHAM820102	Free energy of solution in water, kcal/mole (Charton-Charton, 1982)
314	AURR980110	Membrane-buried preference parameters (Argos et al., 1982)
315	MEIH800102	Average reduced distance for side chain (Meirovitch et al., 1980)
316	NAKH920106	AA composition of CYT of multi-spanning proteins (Nakashima-Nishikawa,
317	RADA880102	Transfer free energy from oct to wat (Radzicka-Wolfenden, 1988)
318	AURR980115	Membrane-buried preference parameters (Argos et al., 1982)
319	CRAJ730101	Normalized frequency of middle helix (Crawford et al., 1973)
320	LAWE840101	Transfer free energy, CHP/water (Lawson et al., 1984)
321	TANS770107	Normalized frequency of left-handed helix (Tanaka-Scheraga, 1977)
322	NAGK730103	Normalized frequency of coil (Nagano, 1973)
323	ROSM880102	Side chain hydropathy, corrected for solvation (Roseman, 1988)
324	MONM990201	Effective partition energy (Miyazawa-Jernigan, 1985)
325	FUKS010101	Helix termination parameter at posision j+1 (Finkelstein et al., 1991)
326	QIAN880112	Weights for alpha-helix at the window position of 5 (Qian-Sejnowski, 19
327	BULH740102	Apparent partial specific volume (Bull-Breese, 1974)
328	FASG760105	pK-C (Fasman, 1976)
329	FAUJ880108	Localized electrical effect (Fauchere et al., 1988)
330	PALJ810105	Normalized frequency of turn from LG (Palau et al., 1981)
331	RICJ880101	Relative preference value at N" (Richardson-Richardson, 1988)
332	RICJ880102	Relative preference value at N' (Richardson-Richardson, 1988)
333	TANS770108	Normalized frequency of zeta R (Tanaka-Scheraga, 1977)
334	AURR980116	Membrane-buried preference parameters (Argos et al., 1982)
335	HUTJ700101	Heat capacity (Hutchens, 1970)
336	GUYH850102	Partition energy (Guy, 1985)
337	TANS770106	Normalized frequency of chain reversal D (Tanaka-Scheraga, 1977)
338	CHOP780212	Frequency of the 1st residue in turn (Chou-Fasman, 1978b)
339	KANM800104	Average relative probability of inner beta-sheet (Kanehisa-Tsong, 1980)
340	AURR980109	Membrane-buried preference parameters (Argos et al., 1982)
341	MAXF760106	Normalized frequency of alpha region (Maxfield-Scheraga, 1976)

342	ROBB760112	Information measure for coil (Robson-Suzuki, 1976)
343	GEOR030101	Aperiodic indices for alpha/beta-proteins (Geisow-Roberts, 1980)
344	CHOP780206	Normalized frequency of N-terminal non helical region (Chou-Fasman, 197
345	NAKH900102	SD of AA composition of total proteins (Nakashima et al., 1990)
346	ZIMJ680101	Weighted second smallest eigenvalue of the weighted Laplacian matrix (K
347	FUKS010111	Helix termination parameter at posision j+1 (Finkelstein et al., 1991)
348	BURA740102	Normalized frequency of extended structure (Burgess et al., 1974)
349	NAKH900112	Transmembrane regions of mt-proteins (Nakashima et al., 1990)
350	ROBB760105	Information measure for extended (Robson-Suzuki, 1976)
351	MEIH800101	Average reduced distance for C-alpha (Meirovitch et al., 1980)
352	RICJ880108	Relative preference value at N5 (Richardson-Richardson, 1988)
353	TANS770110	Normalized frequency of chain reversal (Tanaka-Scheraga, 1977)
354	YUTK870104	Linker propensity from non-helical (annotated by DSSP) dataset (George
355	QIAN880106	Weights for alpha-helix at the window position of -1 (Qian-Sejnowski, 1
356	KANM800103	Average relative probability of inner helix (Kanehisa-Tsong, 1980)
357	LEVM760104	Side chain torsion angle phi(AAAR) (Levitt, 1976)
358	NAKH900111	Transmembrane regions of non-mt-proteins (Nakashima et al., 1990)
359	VHEG790101	Transfer free energy to lipophilic phase (von Heijne-Blomberg, 1979)
360	ZIMJ680104	Weighted second smallest eigenvalue of the weighted Laplacian matrix (K
361	BASU050103	Membrane-buried preference parameters (Argos et al., 1982)
362	WARP780101	Average interactions per side chain atom (Warme-Morgan, 1978)
363	QIAN880136	Weights for coil at the window position of 3 (Qian-Sejnowski, 1988)
364	RACS770102	Average reduced distance for side chain (Rackovsky-Scheraga, 1977)
365	TANS770102	Normalized frequency of isolated helix (Tanaka-Scheraga, 1977)
366	FUKS010106	Helix termination parameter at posision j+1 (Finkelstein et al., 1991)
367	DAYM780201	Relative mutability (Dayhoff et al., 1978b)
368	ISOY800106	Normalized relative frequency of helix end (Isogai et al., 1980)
369	PONP800107	Accessibility reduction ratio (Ponnuswamy et al., 1980)
370	QIAN880101	Weights for alpha-helix at the window position of -6 (Qian-Sejnowski, 1
371	QIAN880134	Weights for coil at the window position of 1 (Qian-Sejnowski, 1988)
372	MUNV940101	Effective partition energy (Miyazawa-Jernigan, 1985)
373	FINA910102	Helix initiation parameter at posision i,i+1,i+2 (Finkelstein et al., 1
374	NAKH900110	Normalized composition of membrane proteins (Nakashima et al., 1990)
375	RACS820110	Average relative fractional occurrence in EL(i-1) (Rackovsky-Scheraga,
376	FAUJ880111	Positive charge (Fauchere et al., 1988)
377	GEIM800111	Aperiodic indices for alpha/beta-proteins (Geisow-Roberts, 1980)
378	PALJ810101	Normalized frequency of alpha-helix from LG (Palau et al., 1981)
379	QIAN880117	Weights for beta-sheet at the window position of -3 (Qian-Sejnowski, 19
380	GUYH850101	Partition energy (Guy, 1985)
381	ROBB760108	Information measure for turn (Robson-Suzuki, 1976)
382	ZIMJ680105	Weighted second smallest eigenvalue of the weighted Laplacian matrix (K
383	CRAJ730102	Normalized frequency of beta-sheet (Crawford et al., 1973)
384	LEVM760103	Side chain angle theta(AAR) (Levitt, 1976)
385	QIAN880104	Weights for alpha-helix at the window position of -3 (Qian-Sejnowski, 1
386	NAKH920101	AA composition of CYT of single-spanning proteins (Nakashima-Nishikawa,
387	QIAN880119	Weights for beta-sheet at the window position of -1 (Qian-Sejnowski, 19
388	QIAN880124	Weights for beta-sheet at the window position of 4 (Qian-Sejnowski, 198
389	RACS820108	Average relative fractional occurrence in AR(i-1) (Rackovsky-Scheraga,
390	RACS820111	Average relative fractional occurrence in E0(i-1) (Rackovsky-Scheraga,

391	FAUJ880107	N.m.r. chemical shift of alpha-carbon (Fauchere et al., 1988)
392	BEGF750101	Conformational parameter of inner helix (Beghin-Dirkx, 1975)
393	CIDH920104	Normalized hydrophobicity scales for alpha/beta-proteins (Cid et al., 1
394	CHAM830101	The Chou-Fasman parameter of the coil conformation (Charton-Charton, 19
395	NAKH900107	AA composition of mt-proteins from fungi and plant (Nakashima et al., 1
396	QIAN880113	Weights for alpha-helix at the window position of 6 (Qian-Sejnowski, 19
397	FUKS010105	Helix termination parameter at posision j+1 (Finkelstein et al., 1991)
398	PRAM820102	Slope in regression analysis x 1.0E1 (Prabhakaran-Ponnuswamy, 1982)
399	RACS820103	Average relative fractional occurrence in AL(i) (Rackovsky-Scheraga, 19
400	ROSM880103	Loss of Side chain hydropathy by helix formation (Roseman, 1988)
401	WILM950104	Linker propensity from non-helical (annotated by DSSP) dataset (George
402	GEOR030105	Aperiodic indices for alpha/beta-proteins (Geisow-Roberts, 1980)
403	QIAN880108	Weights for alpha-helix at the window position of 1 (Qian-Sejnowski, 19
404	AURR980113	Membrane-buried preference parameters (Argos et al., 1982)
405	BURA740101	Normalized frequency of alpha-helix (Burgess et al., 1974)
406	MAXF760102	Normalized frequency of extended structure (Maxfield-Scheraga, 1976)
407	QIAN880138	Weights for coil at the window position of 5 (Qian-Sejnowski, 1988)
408	MUNV940103	Effective partition energy (Miyazawa-Jernigan, 1985)
409	CHAM810101	Steric parameter (Charton, 1981)
410	AURR980103	Membrane-buried preference parameters (Argos et al., 1982)
411	QIAN880133	Weights for coil at the window position of 0 (Qian-Sejnowski, 1988)
412	ROBB760113	Information measure for loop (Robson-Suzuki, 1976)
413	AURR980120	Membrane-buried preference parameters (Argos et al., 1982)
414	NAKH900106	Normalized composition from animal (Nakashima et al., 1990)
415	QIAN880114	Weights for beta-sheet at the window position of -6 (Qian-Sejnowski, 19
416	GEOR030104	Aperiodic indices for alpha/beta-proteins (Geisow-Roberts, 1980)
417	CHAM830103	The number of atoms in the side chain labelled 1+1 (Charton-Charton, 19
418	QIAN880111	Weights for alpha-helix at the window position of 4 (Qian-Sejnowski, 19
419	TANS770103	Normalized frequency of extended structure (Tanaka-Scheraga, 1977)
420	CHOC760104	Proportion of residues 100% buried (Chothia, 1976)
421	DAYM780101	Amino acid composition (Dayhoff et al., 1978a)
422	GEIM800102	Alpha-helix indices for alpha-proteins (Geisow-Roberts, 1980)
423	FAUJ830101	Hydrophobic parameter pi (Fauchere-Pliska, 1983)
424	CEDJ970103	Normalized frequency of extended structure (Burgess et al., 1974)
425	FASG760103	Optical rotation (Fasman, 1976)
426	ISOY800105	Normalized relative frequency of bend S (Isogai et al., 1980)
427	RACS820101	Average relative fractional occurrence in A0(i) (Rackovsky-Scheraga, 19
428	BLAS910101	Information value for accessibility; average fraction 23% (Biou et al.,
429	CHOP780204	Normalized frequency of N-terminal helix (Chou-Fasman, 1978b)
430	PALJ810106	Normalized frequency of turn from CF (Palau et al., 1981)
431	CHOP780101	Normalized frequency of beta-turn (Chou-Fasman, 1978a)
432	GEIM800109	Aperiodic indices for alpha-proteins (Geisow-Roberts, 1980)
433	QIAN880137	Weights for coil at the window position of 4 (Qian-Sejnowski, 1988)
434	QIAN880109	Weights for alpha-helix at the window position of 2 (Qian-Sejnowski, 19
435	NAKH920107	AA composition of EXT of multi-spanning proteins (Nakashima-Nishikawa,
436	PALJ810108	Normalized frequency of alpha-helix in alpha+beta class (Palau et al.,
437	QIAN880103	Weights for alpha-helix at the window position of -4 (Qian-Sejnowski, 1
438	ISOY800104	Normalized relative frequency of bend R (Isogai et al., 1980)
439	QIAN880102	Weights for alpha-helix at the window position of -5 (Qian-Sejnowski, 1

440	PALJ810102	Normalized frequency of alpha-helix from CF (Palau et al., 1981)
441	QIAN880105	Weights for alpha-helix at the window position of -2 (Qian-Sejnowski, 1
442	ROBB760109	Information measure for N-terminal turn (Robson-Suzuki, 1976)
443	TANS770101	Normalized frequency of alpha-helix (Tanaka-Scheraga, 1977)
444	FAUJ880102	Smoothed upsilon steric parameter (Fauchere et al., 1988)
445	NAKH900108	Normalized composition from fungi and plant (Nakashima et al., 1990)
446	QIAN880116	Weights for beta-sheet at the window position of -4 (Qian-Sejnowski, 19
447	NAKH920104	AA composition of EXT2 of single-spanning proteins (Nakashima-Nishikawa
448	FUKS010107	Helix termination parameter at posision j+1 (Finkelstein et al., 1991)
449	ROBB760102	Information measure for N-terminal helix (Robson-Suzuki, 1976)
450	AURR980117	Membrane-buried preference parameters (Argos et al., 1982)
451	NAKH900109	AA composition of membrane proteins (Nakashima et al., 1990)
452	QIAN880121	Weights for beta-sheet at the window position of 1 (Qian-Sejnowski, 198
453	RACS820109	Average relative fractional occurrence in AL(i-1) (Rackovsky-Scheraga,
454	KOEP990101	Net charge (Klein et al., 1984)
455	RACS820113	Value of theta(i) (Rackovsky-Scheraga, 1982)
456	AURR980118	Membrane-buried preference parameters (Argos et al., 1982)
457	GEOR030106	Aperiodic indices for alpha/beta-proteins (Geisow-Roberts, 1980)
458	COHE430101	Partial specific volume (Cohn-Edsall, 1943)
459	RICJ880110	Relative preference value at C5 (Richardson-Richardson, 1988)
460	KUMS000101	Side chain volume (Krigbaum-Komoriya, 1979)
461	EISD860103	Direction of hydrophobic moment (Eisenberg-McLachlan, 1986)
462	AURR980111	Membrane-buried preference parameters (Argos et al., 1982)
463	KOEP990102	Net charge (Klein et al., 1984)
464	CRAJ730103	Normalized frequency of turn (Crawford et al., 1973)
465	YUTK870101	Linker propensity from non-helical (annotated by DSSP) dataset (George
466	GEOR030107	Aperiodic indices for alpha/beta-proteins (Geisow-Roberts, 1980)
467	QIAN880132	Weights for coil at the window position of -1 (Qian-Sejnowski, 1988)
468	QIAN880123	Weights for beta-sheet at the window position of 3 (Qian-Sejnowski, 198
469	ROBB760104	Information measure for C-terminal helix (Robson-Suzuki, 1976)
470	KUMS000102	Side chain volume (Krigbaum-Komoriya, 1979)
471	RICJ880106	Relative preference value at N3 (Richardson-Richardson, 1988)
472	WERD780102	Linker propensity from non-helical (annotated by DSSP) dataset (George
473	WILM950103	Linker propensity from non-helical (annotated by DSSP) dataset (George
474	TANS770109	Normalized frequency of coil (Tanaka-Scheraga, 1977)
475	VELV850101	Electron-ion interaction potential (Veljkovic et al., 1985)
476	COSI940101	Partial specific volume (Cohn-Edsall, 1943)
477	CHOC760103	Proportion of residues 95% buried (Chothia, 1976)
478	QIAN880127	Weights for coil at the window position of -6 (Qian-Sejnowski, 1988)
479	QIAN880135	Weights for coil at the window position of 2 (Qian-Sejnowski, 1988)
480	RICJ880115	Relative preference value at C-cap (Richardson-Richardson, 1988)
481	GEOR030109	Aperiodic indices for alpha/beta-proteins (Geisow-Roberts, 1980)
482	PUNT030102	Beta-coil equilibrium constant (Ptitsyn-Finkelstein, 1983)
483	OLSK800101	Transfer energy, organic solvent/water (Nozaki-Tanford, 1971)
484	JUNJ780101	Sequence frequency (Jungck, 1978)
485	ROBB760101	Information measure for alpha-helix (Robson-Suzuki, 1976)
486	AURR980108	Membrane-buried preference parameters (Argos et al., 1982)
487	BLAM930101	Information value for accessibility; average fraction 23% (Biou et al.,
488	PTIO830101	Helix-coil equilibrium constant (Ptitsyn-Finkelstein, 1983)

489	TANS770105	Normalized frequency of chain reversal S (Tanaka-Scheraga, 1977)
490	CHAM830108	A parameter of charge transfer donor capability (Charton-Charton, 1983)
491	NAGK730102	Normalized frequency of bata-structure (Nagano, 1973)
492	RACS820106	Average relative fractional occurrence in ER(i) (Rackovsky-Scheraga, 19
493	ONEK900101	Transfer energy, organic solvent/water (Nozaki-Tanford, 1971)
494	MAXF760104	Normalized frequency of left-handed alpha-helix (Maxfield-Scheraga, 197
495	OOBM850105	Optimized side chain interaction parameter (Oobatake et al., 1985)
496	JUKT750101	Amino acid distribution (Jukes et al., 1975)
497	AURR980107	Membrane-buried preference parameters (Argos et al., 1982)
498	FINA910103	Helix termination parameter at posision j-2,j-1,j (Finkelstein et al.,
499	ISOY800108	Normalized relative frequency of coil (Isogai et al., 1980)
500	MUNV940105	Effective partition energy (Miyazawa-Jernigan, 1985)
501	FAUJ880113	pK-a(RCOOH) (Fauchere et al., 1988)
502	CHAM830102	A parameter defined from the residuals obtained from the best correlati
503	CHOP780201	Normalized frequency of alpha-helix (Chou-Fasman, 1978b)
504	GEIM800104	Alpha-helix indices for alpha/beta-proteins (Geisow-Roberts, 1980)
505	ISOY800107	Normalized relative frequency of double bend (Isogai et al., 1980)
506	FUKS010112	Helix termination parameter at posision j+1 (Finkelstein et al., 1991)
507	ROBB760103	Information measure for middle helix (Robson-Suzuki, 1976)
508	GEOR030102	Aperiodic indices for alpha/beta-proteins (Geisow-Roberts, 1980)
509	CEDJ970102	Normalized frequency of extended structure (Burgess et al., 1974)
510	NAGK730101	Normalized frequency of alpha-helix (Nagano, 1973)
511	PALJ810113	Normalized frequency of turn in all-alpha class (Palau et al., 1981)
512	FAUJ880112	Negative charge (Fauchere et al., 1988)
513	NAKH900104	Normalized composition of mt-proteins (Nakashima et al., 1990)
514	ROBB760111	Information measure for C-terminal turn (Robson-Suzuki, 1976)
515	FAUJ880105	STERIMOL minimum width of the side chain (Fauchere et al., 1988)
516	MAXF760105	Normalized frequency of zeta L (Maxfield-Scheraga, 1976)
517	CEDJ970101	Normalized frequency of extended structure (Burgess et al., 1974)
518	RACS820114	Value of theta(i-1) (Rackovsky-Scheraga, 1982)
519	MUNV940102	Effective partition energy (Miyazawa-Jernigan, 1985)
520	QIAN880129	Weights for coil at the window position of -4 (Qian-Sejnowski, 1988)
521	CHOP780214	Frequency of the 3rd residue in turn (Chou-Fasman, 1978b)
522	QIAN880107	Weights for alpha-helix at the window position of 0 (Qian-Sejnowski, 19
523	CEDJ970104	Normalized frequency of extended structure (Burgess et al., 1974)
524	FUKS010109	Helix termination parameter at posision j+1 (Finkelstein et al., 1991)
525	ONEK900102	Transfer energy, organic solvent/water (Nozaki-Tanford, 1971)
526	JOND920101	Relative frequency of occurrence (Jones et al., 1992)
527	AURR980112	Membrane-buried preference parameters (Argos et al., 1982)
528	NAKH900101	AA composition of total proteins (Nakashima et al., 1990)
529	ROBB760107	Information measure for extended without H-bond (Robson-Suzuki, 1976)
530	AURR980106	Membrane-buried preference parameters (Argos et al., 1982)
531	FUKS010110	Helix termination parameter at posision j+1 (Finkelstein et al., 1991)
